# Supplementary material for: A configural model of expert judgement as a preliminary epidemiological study of injury problems: An application to drowning
Source: PLoS One. 2019 Oct 24;14(10):e0211166. doi: 10.1371/journal.pone.0211166 (PMC6812787; doi:10.1371/journal.pone.0211166)
Supplement: S1 Supporting Information — (DOCX) [file pone.0211166.s001.docx]

**S1 Supporting information**
