# Supplementary material for: A configural model of expert judgement as a preliminary epidemiological study of injury problems: An application to drowning
Source: PLoS One. 2019 Oct 24;14(10):e0211166. doi: 10.1371/journal.pone.0211166 (PMC6812787; doi:10.1371/journal.pone.0211166)
Supplement: S1 Appendix — (DOCX) [file pone.0211166.s002.docx]

**S2 Appendix**

*Questionnaire instructions*

| The first section of the questionnaire contained three instruction pages. On the first page, the DV term ‘getting into difficulty’ was defined as the situation where bathing at a surf beach ‘a person getting into difficulty in the water would normally require assistance from others to get back to the shore safely’. For control of possible confounding factors, the circumstances pertaining to each vignette (scenario) were provided as follows:  *The person:*   - An adult in good general health; - Had not consumed any drugs or alcohol in the previous 24 hours, and; - The person is not subject to the onset of any medical condition including heart attack, stroke, or epileptic seizure.   *The beach* (a photo of this beach type was included):   - Long sandy beach commonly found in Victoria; - Characterised by regularly spaced sand bars separated by channels dug by rip currents flowing seaward; - Not patrolled by lifesavers or lifeguards, and; - No rocky outcrops or reefs in the bathing zone.   *The day:*   - Warm mid-summer day reaching 28 degrees Celsius; - Water temperature normal for summer at 20 degrees Celsius, and; - Very light offshore breeze.   *The activity:*   - The person intends to bathe by wading or swimming at least as far as the outer zone of breaking waves; - The person intends to bathe four times, each time for a half hour, over a six hour period; - The first bathing episode is at high tide, the last would occur at low (ebb) tide; - The person has no floatation devices or surf equipment including a wetsuit, and; - The person feels comfortable to bathe alone. |
| --- |
