# Supplementary material for: A configural model of expert judgement as a preliminary epidemiological study of injury problems: An application to drowning
Source: PLoS One. 2019 Oct 24;14(10):e0211166. doi: 10.1371/journal.pone.0211166 (PMC6812787; doi:10.1371/journal.pone.0211166)
Supplement: S2 Appendix — (PDF) [file pone.0211166.s003.pdf]

S3 – Appendix

Questionnaire example and Latin square ordering

SURF BEACH SPECIALIST SURVEY

BOOKLET 1

Surf beach specialist survey

Thank you for participating in this research project.

In this survey you will be given a number of scenarios and for each one asked to rate the chance of a person getting into difficulty while they are bathing in the water.

A person getting into difficulty while bathing in the water would normally require assistance from others to get back to the shore safely.

The scenarios are given in two sets. Each set should take you about 15 minutes to complete. In between sets there is a 15 minute break. During this time you will be asked some questions about your own surf beach experience.

The following circumstances apply to each of the scenarios:

The person: Adult, over 17 years of age, in good general health (both mental and physical), and holding an average level of fitness. The person has not consumed any alcohol or other drugs in the last 24 hours. During the scenario, the person is not subject to the onset of a medical condition such as a heart attack, stroke, or epileptic seizure.

The beach: The long sandy ocean beach is of the type most commonly found along Victoria's exposed coastline (known as a 'Transverse bar and rip' beach). It is characterised by regularly spaced sand bars separated by channels dug by rip currents flowing seaward. The beach is not patrolled by lifesavers or lifeguards. There are no rocky outcrops or reefs in the bathing zone (please refer to an *archive* photo of this beach in your folder).

The day: It is a warm mid-summer sunny day reaching 28 degrees Celsius (with water temperature normal for summer at 20 degrees Celsius). There is a very light offshore breeze. The person has gone to the beach for the whole day (6 hours) and plans to bathe four times, each time for around a half hour. The person bathes during an ebb (outgoing) tide; with the first time in the water at high tide and the last time in the water at low tide. The person feels comfortable to bathe alone.

The water bathing activity: The person intends to bathe in the water by wading and/ or swimming at least as far as the outer zone of breaking waves and has no flotation devices, wetsuit, nor other swimming aids/ equipment.

The scale to use when rating each scenario:

For each scenario, the scale below is to be used to indicate your assessment of the chance (or likelihood) of the person getting into difficulty in the water during one of their bathes at the surf beach. You should circle clearly on the scale your best estimate - anywhere from '0%' chance to '100%' chance. For example, if you think the chance of the person getting into difficulty in the water is very low, say 1 chance in 10, then you would circle the 10% mark. If you though the person was almost certain to get into difficulty in the water, then you might circle the 90% mark.

Circling a score over 50% means it is more likely than not that the person will get into difficulty in the water, given the particular scenario. Circling a score under 50% means it is more likely that the person will not get into difficulty in the water, given the particular scenario.

You should make your best estimates based on the facts in each scenario.

Example: Having read and thought about a particular scenario, you rate the person as having a moderate (50%) chance of getting into difficulty in the water – you circle the 50% mark as below:

0%	10%	20%	30%	40%	50%	60%	70%	80%	90%	100%
No chance of getting into difficulty	Low chance of getting into difficulty			Moderate chance of getting into difficulty			High chance of getting into difficult			Certain to get into difficulty

Remember that each scenario occurs in the following circumstances:

- The person is an adult with good general health and fitness,
- The beach is long sandy and unpatrolled,
- The tide is outgoing on a warm sunny summer day with just a light wind,
- The person swims and/ or wades at least as far as the outer break zone,
- The person plan to enter the water four times today, each time for a half hour, over a six-hour period, and,
- The person has no flotation aids.

Important:

When completing each scenario, you must not look back to your rating for previous scenarios.

In a swimming pool Jessica is a relatively strong swimmer, being able to swim constantly for over one hour and float as long as required.

The waves today average up to 2 meters (a little over 6 feet on the old scale) at the break zone. In between the short regular sand bars are troughs carrying strong rip currents reaching about 100 meters from the surf zone seaward.

Jessica regularly goes to the beach. She bathed at a surf beach nearly 50 times last year (about twice a week over the summer period).

The chance (or likelihood) of Jessica getting into difficulty in the water during bathing is:

0%	10%	20%	30%	40%	50%	60%	70%	80%	90%	100%
No chance of getting into difficulty		Low chance of getting into difficulty			Moderate chance of getting into difficulty			High chance of getting into difficult		Certain to get into difficulty

22 402

The waves today average 3 meters (almost 10 feet on the old scale) at the break zone. In between the short sand bars at the outer surf zone are deep troughs carrying very strong and fast rip currents extending some 200 meters from the surf zone seaward.

In a swimming pool Matt is a relatively strong swimmer, being able to swim constantly for over one hour and float as long as required.

Matt occasionally goes to the beach. He bathed at a surf beach about 6 times in the least year (once a month in summer).

The chance (or likelihood) of Matt getting into difficulty in the water during bathing is:

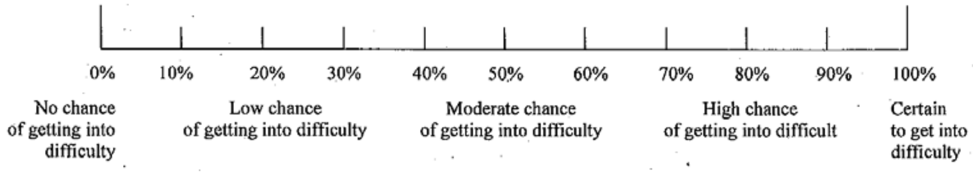

51 606

# Latin square design parameters

Beach specialist risk rating—study plan

1. Risk levels by factors—x (low); y (middle-average); z (high).

A—Swimming competence in a swimming pool

Ax—relatively strong swimmer, s/he can swim constantly for over one hour and float as long as required
Ay—moderately good swimmer, s/he can comfortable float and gently swim for up to 60 minutes
Az—relatively weak swimmer, s/he can comfortable float for at least a minute and gently swim a little way if necessary

B—Surf swimming experience in the last year

Bx—Experienced beach swimmer, s/he swam a surf beach nearly 50 times in the last year (about twice a week over the summer period)
By—Occasional beach swimmer, s/he swam a surf beach about 6 times in the last year (once a month in summer)
Bz—This is the first time s/he has gone swimming in a surf zone at a surf beach

C—Surf conditions—transverse bar beach

Cx—the waves today average 0.5 m (about 1½ feet) at the break zone. In between the long shallow sand bars extending back to the beach are troughs carrying weak to moderate rip currents up to 20 meters past the surf zone. (beach rating 4)
Cy—the waves today average up to 2 m (a little over 6 feet) at the break zone. In between the short regular sand bars are troughs carrying strong rip currents reaching about 100 meters past the surf zone. (beach rating 7)
Cz—the waves today average 3 m (almost 10 feet) at the break zone. In between the short sand bars at the outer surf zone are deep troughs carrying very strong and fast rip currents extending some 200 hundred meters past the surf zone. (beach rating 9)

Possible combinations = 27 (from binomial coefficient)

2. Gender randomisation

Per booklet: Males 13 scenarios
Females 14 scenarios

3. Factor order randomization:

Factor sequence order by gender

Factor order:	Times sequence	Sex (F14/M13)
a (abc)	5	F 3/ M2
b (acb)	5	F 2/M3
c (bac)	5	F 3/M2
d (bca)	4	F 2/M2
e (cab)	4	F 2/M2
f (cba)	4	F 2/M2

4. Vignette risk face value risk score rating

(x=1; y=2; z=3)

**No.**	**Order**	**Risk score**
1 =	(Ax, Bx, Cx)	3
2 =	(Ax, Bx, Cy)	4
3 =	(Ax, Bx, Cz)	5
4 =	(Ax, By, Cx)	4
5 =	(Ax, By, Cy)	5
6 =	(Ax, By, Cz)	6
7 =	(Ax, Bz, Cx)	5
8 =	(Ax, Bz, Cy)	6
9 =	(Ax, Bz, Cz)	7
10 =	(Ay, Bx, Cx)	4
11 =	(Ay, Bx, Cy)	5
12 =	(Ay, Bx, Cz)	6
13 =	(Ay, By, Cx)	7
14 =	(Ay, By, Cy)	6
15 =	(Ay, By, Cz)	7
16 =	(Ay, Bz, Cx)	6
17 =	(Ay, Bz, Cy)	7
18 =	(Ay, Bz, Cz)	8
19 =	(Az, Bx, Cx)	5
20 =	(Az, Bx, Cy)	6
21 =	(Az, Bx, Cz)	7
22 =	(Az, By, Cx)	6
23 =	(Az, By, Cy)	7
24 =	(Az, By, Cz)	8
25 =	(Az, Bz, Cx)	8
26 =	(Az, Bz, Cy)	8
27 =	(Az, Bz, Cz)	9

Latin square order (start point generated by random number order without replacement)

**Gender order:**	**F**	**M**	**F**	**M**	**F**	**M**	**F**	**M**	**F**	**M**	**F**	**M**	**F**	**M**	**F**	**M**	**F**	**M**	**F**	**M**	**F**	**M**	**F**	**M**	**F**	**M**	**F**
**Judge**																											
**A**	7	20	8	19	9	18	10	17	11	16	12	15	13	14	27	1	26	2	25	3	24	4	23	5	22	6	21
**B**	8	21	9	20	10	19	11	18	12	17	13	16	14	15	1	2	27	3	26	4	25	5	24	6	23	7	22
**C**	9	22	10	21	11	20	12	19	13	18	14	17	15	16	2	3	1	4	27	5	26	6	25	7	24	8	23
**D**	10	23	11	22	12	21	13	20	14	19	15	18	16	17	3	4	2	5	1	6	27	7	26	8	25	9	24
**E**	11	24	12	23	13	22	14	21	15	20	16	19	17	18	4	5	3	6	2	7	1	8	27	9	26	10	25
**F**	12	25	13	24	14	23	15	22	16	21	17	20	18	19	5	6	4	7	3	8	2	9	1	10	27	11	26
**G**	13	26	14	25	15	24	16	23	17	22	18	21	19	20	6	7	5	8	4	9	3	10	2	11	1	12	27
**H**	14	27	15	26	16	25	17	24	18	23	19	22	20	21	7	8	6	9	5	10	4	11	3	12	2	13	1
**I**	15	1	16	27	17	26	18	25	19	24	20	23	21	22	8	9	7	10	6	11	5	12	4	13	3	14	2
**J**	16	2	17	1	18	27	19	26	20	25	21	24	22	23	9	10	8	11	7	12	6	13	5	14	4	15	3
**K**	17	3	18	2	19	1	20	27	21	26	22	25	23	24	10	11	9	12	8	13	7	14	6	15	5	16	4
**L**	18	4	19	3	20	2	21	1	22	27	23	26	24	25	11	12	10	13	9	14	8	15	7	16	6	17	5
**M**	19	5	20	4	21	3	22	2	23	1	24	27	25	26	12	13	11	14	10	15	9	16	8	17	7	18	6
**N**	20	6	21	5	22	4	23	3	24	2	25	1	26	27	13	14	12	15	11	16	10	17	9	18	8	19	7
**O**	21	7	22	6	23	5	24	4	25	3	26	2	27	1	14	15	13	16	12	17	11	18	10	19	9	20	8
**P**	22	8	23	7	24	6	25	5	26	4	27	3	1	2	15	16	14	17	13	18	12	19	11	20	10	21	9
**Q**	23	9	24	8	25	7	26	6	27	5	1	4	2	3	16	17	15	18	14	19	13	20	12	21	11	22	10
**R**	24	10	25	9	26	8	27	7	1	6	2	5	3	4	17	18	16	19	15	20	14	21	13	22	12	23	11
**S**	25	11	26	10	27	9	1	8	2	7	3	6	4	5	18	19	17	20	16	21	15	22	14	23	13	24	12
**T**	26	12	27	11	1	10	2	9	3	8	4	7	5	6	19	20	18	21	17	22	16	23	15	24	14	25	13
**U**	27	13	1	12	2	11	3	10	4	9	5	8	6	7	20	21	19	22	18	23	17	24	16	25	15	26	14
**V**	1	14	2	13	3	12	4	11	5	10	6	9	7	8	21	22	20	23	19	24	18	25	17	26	16	27	15
**W**	2	15	3	14	4	13	5	12	6	11	7	10	8	9	22	23	21	24	20	25	19	26	18	27	17	1	16
**X**	3	16	4	15	5	14	6	13	7	12	8	11	9	10	23	24	22	25	21	26	20	27	19	1	18	2	17
**Y**	4	17	5	16	6	15	7	14	8	13	9	12	10	11	24	25	23	26	22	27	21	1	20	2	19	3	18
**Z**	5	18	6	17	7	16	8	15	9	14	10	13	11	12	25	26	24	27	23	1	22	2	21	3	20	4	19
**ZZ**	6	19	7	18	8	17	9	16	10	15	11	14	12	13	26	27	25	1	24	2	23	3	22	4	21	5	20
**Factor order**	**a**	**b**	**c**	**d**	**e**	**f**	**f**	**e**	**d**	**c**	**b**	**a**	**a**	**b**	**c**	**d**	**e**	**f**	**f**	**e**	**d**	**c**	**b**	**a**	**a**	**b**	**c**
