# Supplementary material for: A configural model of expert judgement as a preliminary epidemiological study of injury problems: An application to drowning
Source: PLoS One. 2019 Oct 24;14(10):e0211166. doi: 10.1371/journal.pone.0211166 (PMC6812787; doi:10.1371/journal.pone.0211166)
Supplement: S1 Table — (DOCX) [file pone.0211166.s004.docx]

**S4 Table**

*Judge profiles*

**Specialist judges demographic and beach visitation profile by group.**

|  |  |  | Surfers | | |  | | Lifesavers | |
| --- | --- | --- | --- | --- | --- | --- | --- | --- | --- |
|  |  |  | Case count | % |  | | Case count | | % |
|  |  |  |  |  |  | |  | |  |
| Gender: | |  |  |  |  | |  | |  |
|  | Male |  | 8 | 88.9 |  | | 8 | | 88.9 |
|  | Female |  | 1 | 11.1 |  | | 1 | | 11.1 |
| Age group (years): | |  |  |  |  | |  | |  |
|  | 20-24 |  | 0 | 0.0 |  | | 1 | | 11.1 |
|  | 25-29 |  | 3 | 33.3 |  | | 1 | | 11.1 |
|  | 30-34 |  | 4 | 44.2 |  | | 4 | | 44.2 |
|  | 35-39 |  | 2 | 22.2 |  | | 3 | | 33.3 |
|  |  |  | Mean | SD |  | | Mean | | SD |
| Number of years visited a surf beach: | |  | 23.6 | 8.2 |  | | 25.0 | | 6.1 |
| Times visited a surf beach in previous 12 months: | |  | 177.8 | 97.3 |  | | 91.7 | | 57.1 |

**Specialist judges main surf beach recreational surf-activity profile by group.**

|  | |  | Surfers | |  | Lifesavers | | |
| --- | --- | --- | --- | --- | --- | --- | --- | --- |
|  | |  |  |  |  |  |  | |
|  | |  | Case count | % |  | Case count | % | |
| Main recreational surf-activity: | | |  |  |  |  |  |  |
| Surfing |  | | 9 | 100.0 |  | 2 | 22.2 |  |
| Swimming |  | | 0 | 0.0 |  | 7 | 77.8 |  |
|  |  | |  |  |  |  |  |  |
| Conditions (waves) experience in surf-activity: | | | | |  |  |  |  |
| 2 m | Yes | | 9 | 100.0 |  | 9 | 100.0 |  |
|  | No | | 0 | 0.0 |  | 0 | 0.0 |  |
|  |  | |  |  |  |  |  |  |
| 3 m | Yes | | 8 | 88.9 |  | 9 | 100.0 |  |
|  | No | | 1 | 11.1 |  | 0 | 0.0 |  |
|  |  | |  |  |  |  |  |  |
| Competent to do surf-activity in 3 m waves: | | | |  |  |  |  |  |
|  | Yes | | 8 | 88.9 |  | 9 | 100.0 |  |
|  | No | | 1 | 11.1 |  | 0 | 0.0 |  |
|  |  | |  |  |  |  |  |  |
| Rated surf-activity competence: | | |  |  |  |  |  |  |
| Novice |  | | 0 | 0.0 |  | 0 | 0.0 |  |
| Intermediate |  | | 1 | 11.1 |  | 0 | 0.0 |  |
| Proficient |  | | 6 | 66.7 |  | 3 | 33.3 |  |
| Expert |  | | 2 | 22.2 |  | 6 | 66.7 |  |
|  |  | |  |  |  |  |  | |
|  |  | | Mean | SD |  | Mean | SD | |
| Years experience in the surf-activity: |  | | 18.4 | 6.0 |  | 23.3 | 5.7 | |
|  |  | |  |  |  |  |  | |
| Surf-activity participation in previous 12 months (frequency): | | | 113.3 | 68.4 |  | 70.6 | 57.4 | |

**Specialist judges qualifications and life saving experience by group.**

|  | |  | Surfers | | | |  | Lifesavers | | | |
| --- | --- | --- | --- | --- | --- | --- | --- | --- | --- | --- | --- |
|  | |  | Case count | | | |  | Case count | | | |
| Self-reported Qualifications: | |  |  | |  | |  |  | |  | |
| Surf related | |  | 1 | | | |  | 9 | | | |
| Swimming | |  | 4 | | | |  | 7 | | | |
| First aid | |  | 5 | | | |  | 9 | | | |
|  | |  |  | |  | |  |  | |  | |
| Rescue experience: |  | | | |  | |  |  | |  | |
| Rescues performed | |  | 4 | | | |  | 9 | | | |
| CPR administered | |  | 0 | | | |  | 6 | | | |
| Patient hospitalised | |  | 0 | | | |  | 9 | | | |
|  | |  |  |  | | |  |  |  | | |
|  | |  | Mean | | | SD |  | Mean | | | SD |
| Lifesaving years: | |  |  | | |  |  |  | | |  |
| Voluntary | |  | 0.0 | | | 0.0 |  | 14.0 | | | 5.6 |
| Professional | |  | 0.0 | | | 0.0 |  | 6.4 | | | 6.7 |
| Total | |  | 0.0 | | | 0.0 |  | 19.9 | | | 10.4 |
|  | |  |  | | |  |  |  | | |  |
| Rescues: | |  |  | | |  |  |  | | |  |
| Total performed | |  | 2.3 | | | 3.0 |  | 270.6 | | | 223.8 |
| CPR administered | |  | 0.0 | | | 0.0 |  | 1.4 | | | 1.2 |
| Patients hospitalised | |  | 0.0 | | | 0.0 |  | 13.9 | | | 13.3 |
