# Supplementary material for: A configural model of expert judgement as a preliminary epidemiological study of injury problems: An application to drowning
Source: PLoS One. 2019 Oct 24;14(10):e0211166. doi: 10.1371/journal.pone.0211166 (PMC6812787; doi:10.1371/journal.pone.0211166)

**S5 Data output**

GET

FILE='C:\Users\damianm\2018\Research\Configural judgement paper\PLOS\Vig means used final final hopefully.sav'.

DATASET NAME DataSet1 WINDOW=FRONT.

SAVE OUTFILE='C:\Users\damianm\2018\Specialist ratings.sav'

/COMPRESSED.

DATASET ACTIVATE DataSet1.

SAVE OUTFILE='C:\Users\damianm\2018\Specialist ratings.sav'

/COMPRESSED.

*Nonparametric Tests: Independent Samples.

NPTESTS

/INDEPENDENT TEST (V1.A1.B1.C1) GROUP (V1.sex) KOLMOGOROV_SMIRNOV

/MISSING SCOPE=ANALYSIS USERMISSING=EXCLUDE

/CRITERIA ALPHA=0.05 CILEVEL=95.

**Nonparametric Tests**

| **Notes** | | |
| --- | --- | --- |
| Output Created | | 13-SEP-2019 08:45:09 |
| Comments | |  |
| Input | Data | C:\Users\damianm\2018\Specialist ratings.sav |
|  | Active Dataset | DataSet1 |
|  | Filter | <none> |
|  | Weight | <none> |
|  | Split File | <none> |
|  | N of Rows in Working Data File | 36 |
| Syntax | | NPTESTS  /INDEPENDENT TEST (V1.A1.B1.C1) GROUP (V1.sex) KOLMOGOROV_SMIRNOV  /MISSING SCOPE=ANALYSIS USERMISSING=EXCLUDE  /CRITERIA ALPHA=0.05 CILEVEL=95. |
| Resources | Processor Time | 00:00:00.28 |
|  | Elapsed Time | 00:00:00.31 |

[DataSet1] C:\Users\damianm\2018\Specialist ratings.sav

**null : null**


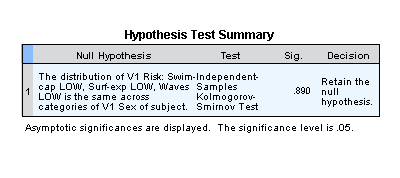


*Nonparametric Tests: Independent Samples.

NPTESTS

/INDEPENDENT TEST (V2.A1.B1.C2) GROUP (V2.sex) KOLMOGOROV_SMIRNOV

/MISSING SCOPE=ANALYSIS USERMISSING=EXCLUDE

/CRITERIA ALPHA=0.05 CILEVEL=95.

**Nonparametric Tests**

| **Notes** | | |
| --- | --- | --- |
| Output Created | | 13-SEP-2019 08:47:49 |
| Comments | |  |
| Input | Data | C:\Users\damianm\2018\Specialist ratings.sav |
|  | Active Dataset | DataSet1 |
|  | Filter | <none> |
|  | Weight | <none> |
|  | Split File | <none> |
|  | N of Rows in Working Data File | 36 |
| Syntax | | NPTESTS  /INDEPENDENT TEST (V2.A1.B1.C2) GROUP (V2.sex) KOLMOGOROV_SMIRNOV  /MISSING SCOPE=ANALYSIS USERMISSING=EXCLUDE  /CRITERIA ALPHA=0.05 CILEVEL=95. |
| Resources | Processor Time | 00:00:00.36 |
|  | Elapsed Time | 00:00:00.20 |

**null : null**


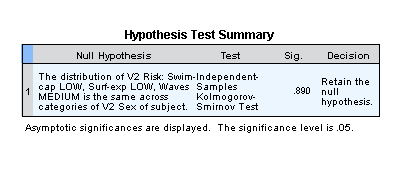


*Nonparametric Tests: Independent Samples.

NPTESTS

/INDEPENDENT TEST (V3.A1.B1.C3) GROUP (V3.sex) KOLMOGOROV_SMIRNOV

/MISSING SCOPE=ANALYSIS USERMISSING=EXCLUDE

/CRITERIA ALPHA=0.05 CILEVEL=95.

**Nonparametric Tests**

| **Notes** | | |
| --- | --- | --- |
| Output Created | | 13-SEP-2019 08:48:31 |
| Comments | |  |
| Input | Data | C:\Users\damianm\2018\Specialist ratings.sav |
|  | Active Dataset | DataSet1 |
|  | Filter | <none> |
|  | Weight | <none> |
|  | Split File | <none> |
|  | N of Rows in Working Data File | 36 |
| Syntax | | NPTESTS  /INDEPENDENT TEST (V3.A1.B1.C3) GROUP (V3.sex) KOLMOGOROV_SMIRNOV  /MISSING SCOPE=ANALYSIS USERMISSING=EXCLUDE  /CRITERIA ALPHA=0.05 CILEVEL=95. |
| Resources | Processor Time | 00:00:00.27 |
|  | Elapsed Time | 00:00:00.19 |

**null : null**


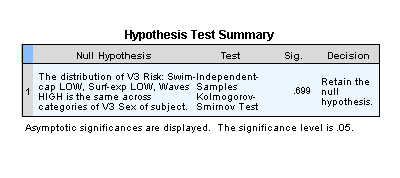


*Nonparametric Tests: Independent Samples.

NPTESTS

/INDEPENDENT TEST (V4.A1.B2.C1) GROUP (V4.sex) KOLMOGOROV_SMIRNOV

/MISSING SCOPE=ANALYSIS USERMISSING=EXCLUDE

/CRITERIA ALPHA=0.05 CILEVEL=95.

**Nonparametric Tests**

| **Notes** | | |
| --- | --- | --- |
| Output Created | | 13-SEP-2019 08:50:22 |
| Comments | |  |
| Input | Data | C:\Users\damianm\2018\Specialist ratings.sav |
|  | Active Dataset | DataSet1 |
|  | Filter | <none> |
|  | Weight | <none> |
|  | Split File | <none> |
|  | N of Rows in Working Data File | 36 |
| Syntax | | NPTESTS  /INDEPENDENT TEST (V4.A1.B2.C1) GROUP (V4.sex) KOLMOGOROV_SMIRNOV  /MISSING SCOPE=ANALYSIS USERMISSING=EXCLUDE  /CRITERIA ALPHA=0.05 CILEVEL=95. |
| Resources | Processor Time | 00:00:00.23 |
|  | Elapsed Time | 00:00:00.17 |

**null : null**


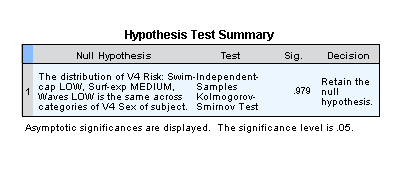


*Nonparametric Tests: Independent Samples.

NPTESTS

/INDEPENDENT TEST (V5.A1.B2.C2) GROUP (V5.sex) KOLMOGOROV_SMIRNOV

/MISSING SCOPE=ANALYSIS USERMISSING=EXCLUDE

/CRITERIA ALPHA=0.05 CILEVEL=95.

**Nonparametric Tests**

| **Notes** | | |
| --- | --- | --- |
| Output Created | | 13-SEP-2019 08:50:58 |
| Comments | |  |
| Input | Data | C:\Users\damianm\2018\Specialist ratings.sav |
|  | Active Dataset | DataSet1 |
|  | Filter | <none> |
|  | Weight | <none> |
|  | Split File | <none> |
|  | N of Rows in Working Data File | 36 |
| Syntax | | NPTESTS  /INDEPENDENT TEST (V5.A1.B2.C2) GROUP (V5.sex) KOLMOGOROV_SMIRNOV  /MISSING SCOPE=ANALYSIS USERMISSING=EXCLUDE  /CRITERIA ALPHA=0.05 CILEVEL=95. |
| Resources | Processor Time | 00:00:00.28 |
|  | Elapsed Time | 00:00:00.29 |

**null : null**


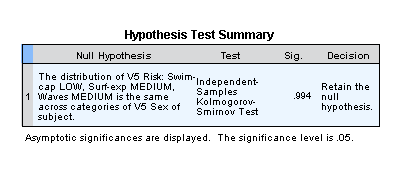


*Nonparametric Tests: Independent Samples.

NPTESTS

/INDEPENDENT TEST (V6.A1.B2.C3) GROUP (V6.sex) KOLMOGOROV_SMIRNOV

/MISSING SCOPE=ANALYSIS USERMISSING=EXCLUDE

/CRITERIA ALPHA=0.05 CILEVEL=95.

**Nonparametric Tests**

| **Notes** | | |
| --- | --- | --- |
| Output Created | | 13-SEP-2019 08:51:49 |
| Comments | |  |
| Input | Data | C:\Users\damianm\2018\Specialist ratings.sav |
|  | Active Dataset | DataSet1 |
|  | Filter | <none> |
|  | Weight | <none> |
|  | Split File | <none> |
|  | N of Rows in Working Data File | 36 |
| Syntax | | NPTESTS  /INDEPENDENT TEST (V6.A1.B2.C3) GROUP (V6.sex) KOLMOGOROV_SMIRNOV  /MISSING SCOPE=ANALYSIS USERMISSING=EXCLUDE  /CRITERIA ALPHA=0.05 CILEVEL=95. |
| Resources | Processor Time | 00:00:00.31 |
|  | Elapsed Time | 00:00:00.19 |

**null : null**


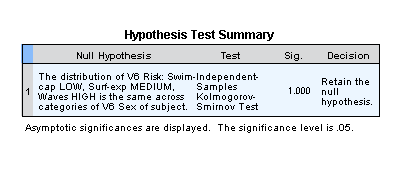


*Nonparametric Tests: Independent Samples.

NPTESTS

/INDEPENDENT TEST (V7.A1.B3.C1) GROUP (V7.sex) KOLMOGOROV_SMIRNOV

/MISSING SCOPE=ANALYSIS USERMISSING=EXCLUDE

/CRITERIA ALPHA=0.05 CILEVEL=95.

**Nonparametric Tests**

| **Notes** | | |
| --- | --- | --- |
| Output Created | | 13-SEP-2019 08:52:35 |
| Comments | |  |
| Input | Data | C:\Users\damianm\2018\Specialist ratings.sav |
|  | Active Dataset | DataSet1 |
|  | Filter | <none> |
|  | Weight | <none> |
|  | Split File | <none> |
|  | N of Rows in Working Data File | 36 |
| Syntax | | NPTESTS  /INDEPENDENT TEST (V7.A1.B3.C1) GROUP (V7.sex) KOLMOGOROV_SMIRNOV  /MISSING SCOPE=ANALYSIS USERMISSING=EXCLUDE  /CRITERIA ALPHA=0.05 CILEVEL=95. |
| Resources | Processor Time | 00:00:00.36 |
|  | Elapsed Time | 00:00:00.21 |

**null : null**


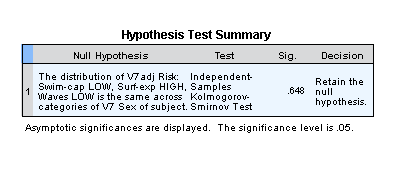


*Nonparametric Tests: Independent Samples.

NPTESTS

/INDEPENDENT TEST (V8.A1.B3.C2) GROUP (V8.sex) KOLMOGOROV_SMIRNOV

/MISSING SCOPE=ANALYSIS USERMISSING=EXCLUDE

/CRITERIA ALPHA=0.05 CILEVEL=95.

**Nonparametric Tests**

| **Notes** | | |
| --- | --- | --- |
| Output Created | | 13-SEP-2019 08:52:59 |
| Comments | |  |
| Input | Data | C:\Users\damianm\2018\Specialist ratings.sav |
|  | Active Dataset | DataSet1 |
|  | Filter | <none> |
|  | Weight | <none> |
|  | Split File | <none> |
|  | N of Rows in Working Data File | 36 |
| Syntax | | NPTESTS  /INDEPENDENT TEST (V8.A1.B3.C2) GROUP (V8.sex) KOLMOGOROV_SMIRNOV  /MISSING SCOPE=ANALYSIS USERMISSING=EXCLUDE  /CRITERIA ALPHA=0.05 CILEVEL=95. |
| Resources | Processor Time | 00:00:00.25 |
|  | Elapsed Time | 00:00:00.25 |

**null : null**


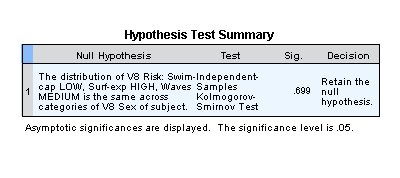


*Nonparametric Tests: Independent Samples.

NPTESTS

/INDEPENDENT TEST (V9.A1.B3.C3) GROUP (V9.sex) KOLMOGOROV_SMIRNOV

/MISSING SCOPE=ANALYSIS USERMISSING=EXCLUDE

/CRITERIA ALPHA=0.05 CILEVEL=95.

**Nonparametric Tests**

| **Notes** | | |
| --- | --- | --- |
| Output Created | | 13-SEP-2019 08:53:21 |
| Comments | |  |
| Input | Data | C:\Users\damianm\2018\Specialist ratings.sav |
|  | Active Dataset | DataSet1 |
|  | Filter | <none> |
|  | Weight | <none> |
|  | Split File | <none> |
|  | N of Rows in Working Data File | 36 |
| Syntax | | NPTESTS  /INDEPENDENT TEST (V9.A1.B3.C3) GROUP (V9.sex) KOLMOGOROV_SMIRNOV  /MISSING SCOPE=ANALYSIS USERMISSING=EXCLUDE  /CRITERIA ALPHA=0.05 CILEVEL=95. |
| Resources | Processor Time | 00:00:00.13 |
|  | Elapsed Time | 00:00:00.19 |

**null : null**


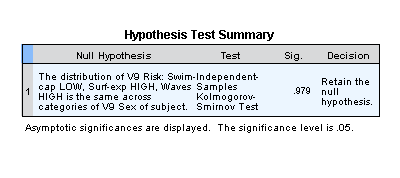


*Nonparametric Tests: Independent Samples.

NPTESTS

/INDEPENDENT TEST (V10.A2.B1.C1) GROUP (V10.sex) KOLMOGOROV_SMIRNOV

/MISSING SCOPE=ANALYSIS USERMISSING=EXCLUDE

/CRITERIA ALPHA=0.05 CILEVEL=95.

**Nonparametric Tests**

| **Notes** | | |
| --- | --- | --- |
| Output Created | | 13-SEP-2019 08:53:42 |
| Comments | |  |
| Input | Data | C:\Users\damianm\2018\Specialist ratings.sav |
|  | Active Dataset | DataSet1 |
|  | Filter | <none> |
|  | Weight | <none> |
|  | Split File | <none> |
|  | N of Rows in Working Data File | 36 |
| Syntax | | NPTESTS  /INDEPENDENT TEST (V10.A2.B1.C1) GROUP (V10.sex) KOLMOGOROV_SMIRNOV  /MISSING SCOPE=ANALYSIS USERMISSING=EXCLUDE  /CRITERIA ALPHA=0.05 CILEVEL=95. |
| Resources | Processor Time | 00:00:00.23 |
|  | Elapsed Time | 00:00:00.15 |

**null : null**


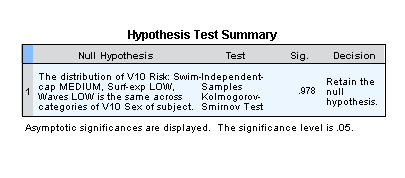


*Nonparametric Tests: Independent Samples.

NPTESTS

/INDEPENDENT TEST (V11.A2.B1.C2) GROUP (V11.sex) KOLMOGOROV_SMIRNOV

/MISSING SCOPE=ANALYSIS USERMISSING=EXCLUDE

/CRITERIA ALPHA=0.05 CILEVEL=95.

**Nonparametric Tests**

| **Notes** | | |
| --- | --- | --- |
| Output Created | | 13-SEP-2019 08:54:03 |
| Comments | |  |
| Input | Data | C:\Users\damianm\2018\Specialist ratings.sav |
|  | Active Dataset | DataSet1 |
|  | Filter | <none> |
|  | Weight | <none> |
|  | Split File | <none> |
|  | N of Rows in Working Data File | 36 |
| Syntax | | NPTESTS  /INDEPENDENT TEST (V11.A2.B1.C2) GROUP (V11.sex) KOLMOGOROV_SMIRNOV  /MISSING SCOPE=ANALYSIS USERMISSING=EXCLUDE  /CRITERIA ALPHA=0.05 CILEVEL=95. |
| Resources | Processor Time | 00:00:00.30 |
|  | Elapsed Time | 00:00:00.17 |

**null : null**


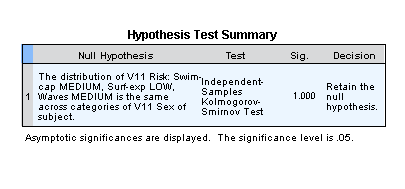


*Nonparametric Tests: Independent Samples.

NPTESTS

/INDEPENDENT TEST (V12.A2.B1.C3) GROUP (V12.sex) KOLMOGOROV_SMIRNOV

/MISSING SCOPE=ANALYSIS USERMISSING=EXCLUDE

/CRITERIA ALPHA=0.05 CILEVEL=95.

**Nonparametric Tests**

| **Notes** | | |
| --- | --- | --- |
| Output Created | | 13-SEP-2019 08:54:23 |
| Comments | |  |
| Input | Data | C:\Users\damianm\2018\Specialist ratings.sav |
|  | Active Dataset | DataSet1 |
|  | Filter | <none> |
|  | Weight | <none> |
|  | Split File | <none> |
|  | N of Rows in Working Data File | 36 |
| Syntax | | NPTESTS  /INDEPENDENT TEST (V12.A2.B1.C3) GROUP (V12.sex) KOLMOGOROV_SMIRNOV  /MISSING SCOPE=ANALYSIS USERMISSING=EXCLUDE  /CRITERIA ALPHA=0.05 CILEVEL=95. |
| Resources | Processor Time | 00:00:00.19 |
|  | Elapsed Time | 00:00:00.15 |

**null : null**


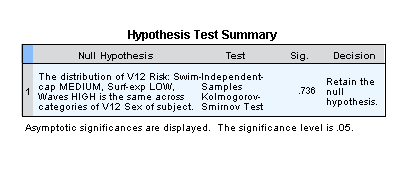


*Nonparametric Tests: Independent Samples.

NPTESTS

/INDEPENDENT TEST (V13.A2.B2.C1) GROUP (V13.sex) KOLMOGOROV_SMIRNOV

/MISSING SCOPE=ANALYSIS USERMISSING=EXCLUDE

/CRITERIA ALPHA=0.05 CILEVEL=95.

**Nonparametric Tests**

| **Notes** | | |
| --- | --- | --- |
| Output Created | | 13-SEP-2019 08:54:46 |
| Comments | |  |
| Input | Data | C:\Users\damianm\2018\Specialist ratings.sav |
|  | Active Dataset | DataSet1 |
|  | Filter | <none> |
|  | Weight | <none> |
|  | Split File | <none> |
|  | N of Rows in Working Data File | 36 |
| Syntax | | NPTESTS  /INDEPENDENT TEST (V13.A2.B2.C1) GROUP (V13.sex) KOLMOGOROV_SMIRNOV  /MISSING SCOPE=ANALYSIS USERMISSING=EXCLUDE  /CRITERIA ALPHA=0.05 CILEVEL=95. |
| Resources | Processor Time | 00:00:00.22 |
|  | Elapsed Time | 00:00:00.15 |

**null : null**


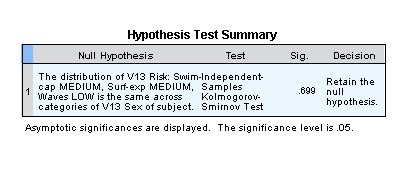


*Nonparametric Tests: Independent Samples.

NPTESTS

/INDEPENDENT TEST (V14.A2.B2.C2) GROUP (V14.sex) KOLMOGOROV_SMIRNOV

/MISSING SCOPE=ANALYSIS USERMISSING=EXCLUDE

/CRITERIA ALPHA=0.05 CILEVEL=95.

**Nonparametric Tests**

| **Notes** | | |
| --- | --- | --- |
| Output Created | | 13-SEP-2019 08:55:42 |
| Comments | |  |
| Input | Data | C:\Users\damianm\2018\Specialist ratings.sav |
|  | Active Dataset | DataSet1 |
|  | Filter | <none> |
|  | Weight | <none> |
|  | Split File | <none> |
|  | N of Rows in Working Data File | 36 |
| Syntax | | NPTESTS  /INDEPENDENT TEST (V14.A2.B2.C2) GROUP (V14.sex) KOLMOGOROV_SMIRNOV  /MISSING SCOPE=ANALYSIS USERMISSING=EXCLUDE  /CRITERIA ALPHA=0.05 CILEVEL=95. |
| Resources | Processor Time | 00:00:00.14 |
|  | Elapsed Time | 00:00:00.14 |

**null : null**


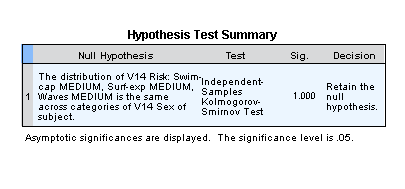


*Nonparametric Tests: Independent Samples.

NPTESTS

/INDEPENDENT TEST (V15.A2.B2.C3) GROUP (V15.sex) KOLMOGOROV_SMIRNOV

/MISSING SCOPE=ANALYSIS USERMISSING=EXCLUDE

/CRITERIA ALPHA=0.05 CILEVEL=95.

**Nonparametric Tests**

| **Notes** | | |
| --- | --- | --- |
| Output Created | | 13-SEP-2019 08:56:02 |
| Comments | |  |
| Input | Data | C:\Users\damianm\2018\Specialist ratings.sav |
|  | Active Dataset | DataSet1 |
|  | Filter | <none> |
|  | Weight | <none> |
|  | Split File | <none> |
|  | N of Rows in Working Data File | 36 |
| Syntax | | NPTESTS  /INDEPENDENT TEST (V15.A2.B2.C3) GROUP (V15.sex) KOLMOGOROV_SMIRNOV  /MISSING SCOPE=ANALYSIS USERMISSING=EXCLUDE  /CRITERIA ALPHA=0.05 CILEVEL=95. |
| Resources | Processor Time | 00:00:00.19 |
|  | Elapsed Time | 00:00:00.18 |

**null : null**


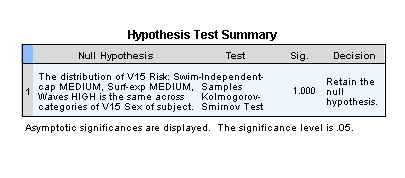


*Nonparametric Tests: Independent Samples.

NPTESTS

/INDEPENDENT TEST (V16.A2.B3.C1) GROUP (V16.sex) KOLMOGOROV_SMIRNOV

/MISSING SCOPE=ANALYSIS USERMISSING=EXCLUDE

/CRITERIA ALPHA=0.05 CILEVEL=95.

**Nonparametric Tests**

| **Notes** | | |
| --- | --- | --- |
| Output Created | | 13-SEP-2019 08:56:23 |
| Comments | |  |
| Input | Data | C:\Users\damianm\2018\Specialist ratings.sav |
|  | Active Dataset | DataSet1 |
|  | Filter | <none> |
|  | Weight | <none> |
|  | Split File | <none> |
|  | N of Rows in Working Data File | 36 |
| Syntax | | NPTESTS  /INDEPENDENT TEST (V16.A2.B3.C1) GROUP (V16.sex) KOLMOGOROV_SMIRNOV  /MISSING SCOPE=ANALYSIS USERMISSING=EXCLUDE  /CRITERIA ALPHA=0.05 CILEVEL=95. |
| Resources | Processor Time | 00:00:00.17 |
|  | Elapsed Time | 00:00:00.14 |

**null : null**


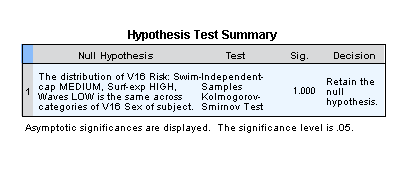


*Nonparametric Tests: Independent Samples.

NPTESTS

/INDEPENDENT TEST (V17.A2.B3.C2) GROUP (V17.sex) KOLMOGOROV_SMIRNOV

/MISSING SCOPE=ANALYSIS USERMISSING=EXCLUDE

/CRITERIA ALPHA=0.05 CILEVEL=95.

**Nonparametric Tests**

| **Notes** | | |
| --- | --- | --- |
| Output Created | | 13-SEP-2019 08:56:48 |
| Comments | |  |
| Input | Data | C:\Users\damianm\2018\Specialist ratings.sav |
|  | Active Dataset | DataSet1 |
|  | Filter | <none> |
|  | Weight | <none> |
|  | Split File | <none> |
|  | N of Rows in Working Data File | 36 |
| Syntax | | NPTESTS  /INDEPENDENT TEST (V17.A2.B3.C2) GROUP (V17.sex) KOLMOGOROV_SMIRNOV  /MISSING SCOPE=ANALYSIS USERMISSING=EXCLUDE  /CRITERIA ALPHA=0.05 CILEVEL=95. |
| Resources | Processor Time | 00:00:00.11 |
|  | Elapsed Time | 00:00:00.17 |

**null : null**


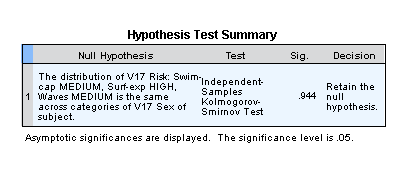


*Nonparametric Tests: Independent Samples.

NPTESTS

/INDEPENDENT TEST (V18.A2.B3.C3) GROUP (V18.sex) KOLMOGOROV_SMIRNOV

/MISSING SCOPE=ANALYSIS USERMISSING=EXCLUDE

/CRITERIA ALPHA=0.05 CILEVEL=95.

**Nonparametric Tests**

| **Notes** | | |
| --- | --- | --- |
| Output Created | | 13-SEP-2019 08:57:05 |
| Comments | |  |
| Input | Data | C:\Users\damianm\2018\Specialist ratings.sav |
|  | Active Dataset | DataSet1 |
|  | Filter | <none> |
|  | Weight | <none> |
|  | Split File | <none> |
|  | N of Rows in Working Data File | 36 |
| Syntax | | NPTESTS  /INDEPENDENT TEST (V18.A2.B3.C3) GROUP (V18.sex) KOLMOGOROV_SMIRNOV  /MISSING SCOPE=ANALYSIS USERMISSING=EXCLUDE  /CRITERIA ALPHA=0.05 CILEVEL=95. |
| Resources | Processor Time | 00:00:00.20 |
|  | Elapsed Time | 00:00:00.18 |

**null : null**


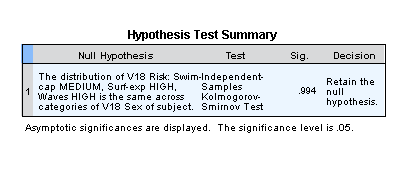


*Nonparametric Tests: Independent Samples.

NPTESTS

/INDEPENDENT TEST (V19.A3.B1.C1) GROUP (V19.sex) KOLMOGOROV_SMIRNOV

/MISSING SCOPE=ANALYSIS USERMISSING=EXCLUDE

/CRITERIA ALPHA=0.05 CILEVEL=95.

**Nonparametric Tests**

| **Notes** | | |
| --- | --- | --- |
| Output Created | | 13-SEP-2019 08:57:23 |
| Comments | |  |
| Input | Data | C:\Users\damianm\2018\Specialist ratings.sav |
|  | Active Dataset | DataSet1 |
|  | Filter | <none> |
|  | Weight | <none> |
|  | Split File | <none> |
|  | N of Rows in Working Data File | 36 |
| Syntax | | NPTESTS  /INDEPENDENT TEST (V19.A3.B1.C1) GROUP (V19.sex) KOLMOGOROV_SMIRNOV  /MISSING SCOPE=ANALYSIS USERMISSING=EXCLUDE  /CRITERIA ALPHA=0.05 CILEVEL=95. |
| Resources | Processor Time | 00:00:00.16 |
|  | Elapsed Time | 00:00:00.13 |

**null : null**


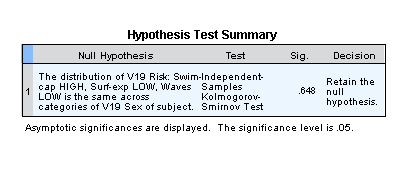


*Nonparametric Tests: Independent Samples.

NPTESTS

/INDEPENDENT TEST (V20.A3.B1.C2) GROUP (V20.sex) KOLMOGOROV_SMIRNOV

/MISSING SCOPE=ANALYSIS USERMISSING=EXCLUDE

/CRITERIA ALPHA=0.05 CILEVEL=95.

**Nonparametric Tests**

| **Notes** | | |
| --- | --- | --- |
| Output Created | | 13-SEP-2019 08:57:46 |
| Comments | |  |
| Input | Data | C:\Users\damianm\2018\Specialist ratings.sav |
|  | Active Dataset | DataSet1 |
|  | Filter | <none> |
|  | Weight | <none> |
|  | Split File | <none> |
|  | N of Rows in Working Data File | 36 |
| Syntax | | NPTESTS  /INDEPENDENT TEST (V20.A3.B1.C2) GROUP (V20.sex) KOLMOGOROV_SMIRNOV  /MISSING SCOPE=ANALYSIS USERMISSING=EXCLUDE  /CRITERIA ALPHA=0.05 CILEVEL=95. |
| Resources | Processor Time | 00:00:00.16 |
|  | Elapsed Time | 00:00:00.16 |

**null : null**


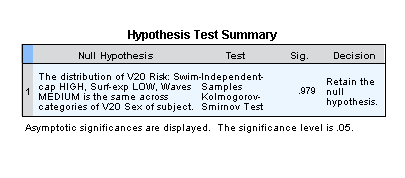


*Nonparametric Tests: Independent Samples.

NPTESTS

/INDEPENDENT TEST (V21.A3.B1.C3) GROUP (V21.sex) KOLMOGOROV_SMIRNOV

/MISSING SCOPE=ANALYSIS USERMISSING=EXCLUDE

/CRITERIA ALPHA=0.05 CILEVEL=95.

**Nonparametric Tests**

| **Notes** | | |
| --- | --- | --- |
| Output Created | | 13-SEP-2019 08:58:22 |
| Comments | |  |
| Input | Data | C:\Users\damianm\2018\Specialist ratings.sav |
|  | Active Dataset | DataSet1 |
|  | Filter | <none> |
|  | Weight | <none> |
|  | Split File | <none> |
|  | N of Rows in Working Data File | 36 |
| Syntax | | NPTESTS  /INDEPENDENT TEST (V21.A3.B1.C3) GROUP (V21.sex) KOLMOGOROV_SMIRNOV  /MISSING SCOPE=ANALYSIS USERMISSING=EXCLUDE  /CRITERIA ALPHA=0.05 CILEVEL=95. |
| Resources | Processor Time | 00:00:00.17 |
|  | Elapsed Time | 00:00:00.15 |

**null : null**


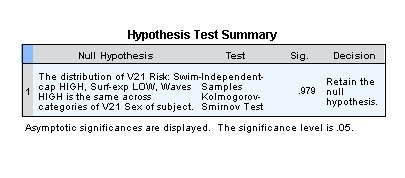


*Nonparametric Tests: Independent Samples.

NPTESTS

/INDEPENDENT TEST (V22.A3.B2.C1) GROUP (V22.sex) KOLMOGOROV_SMIRNOV

/MISSING SCOPE=ANALYSIS USERMISSING=EXCLUDE

/CRITERIA ALPHA=0.05 CILEVEL=95.

**Nonparametric Tests**

| **Notes** | | |
| --- | --- | --- |
| Output Created | | 13-SEP-2019 08:58:50 |
| Comments | |  |
| Input | Data | C:\Users\damianm\2018\Specialist ratings.sav |
|  | Active Dataset | DataSet1 |
|  | Filter | <none> |
|  | Weight | <none> |
|  | Split File | <none> |
|  | N of Rows in Working Data File | 36 |
| Syntax | | NPTESTS  /INDEPENDENT TEST (V22.A3.B2.C1) GROUP (V22.sex) KOLMOGOROV_SMIRNOV  /MISSING SCOPE=ANALYSIS USERMISSING=EXCLUDE  /CRITERIA ALPHA=0.05 CILEVEL=95. |
| Resources | Processor Time | 00:00:00.22 |
|  | Elapsed Time | 00:00:00.17 |

**null : null**


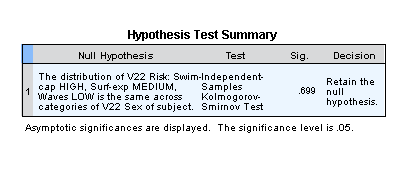


*Nonparametric Tests: Independent Samples.

NPTESTS

/INDEPENDENT TEST (V23.A3.B2.C2) GROUP (V23.sex) KOLMOGOROV_SMIRNOV

/MISSING SCOPE=ANALYSIS USERMISSING=EXCLUDE

/CRITERIA ALPHA=0.05 CILEVEL=95.

**Nonparametric Tests**

| **Notes** | | |
| --- | --- | --- |
| Output Created | | 13-SEP-2019 08:59:07 |
| Comments | |  |
| Input | Data | C:\Users\damianm\2018\Specialist ratings.sav |
|  | Active Dataset | DataSet1 |
|  | Filter | <none> |
|  | Weight | <none> |
|  | Split File | <none> |
|  | N of Rows in Working Data File | 36 |
| Syntax | | NPTESTS  /INDEPENDENT TEST (V23.A3.B2.C2) GROUP (V23.sex) KOLMOGOROV_SMIRNOV  /MISSING SCOPE=ANALYSIS USERMISSING=EXCLUDE  /CRITERIA ALPHA=0.05 CILEVEL=95. |
| Resources | Processor Time | 00:00:00.17 |
|  | Elapsed Time | 00:00:00.15 |

**null : null**


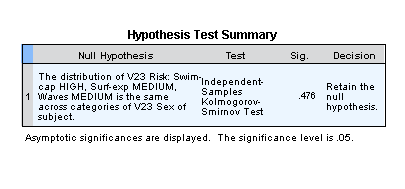


*Nonparametric Tests: Independent Samples.

NPTESTS

/INDEPENDENT TEST (V24.A3.B2.C3) GROUP (V24.sex) KOLMOGOROV_SMIRNOV

/MISSING SCOPE=ANALYSIS USERMISSING=EXCLUDE

/CRITERIA ALPHA=0.05 CILEVEL=95.

**Nonparametric Tests**

| **Notes** | | |
| --- | --- | --- |
| Output Created | | 13-SEP-2019 08:59:28 |
| Comments | |  |
| Input | Data | C:\Users\damianm\2018\Specialist ratings.sav |
|  | Active Dataset | DataSet1 |
|  | Filter | <none> |
|  | Weight | <none> |
|  | Split File | <none> |
|  | N of Rows in Working Data File | 36 |
| Syntax | | NPTESTS  /INDEPENDENT TEST (V24.A3.B2.C3) GROUP (V24.sex) KOLMOGOROV_SMIRNOV  /MISSING SCOPE=ANALYSIS USERMISSING=EXCLUDE  /CRITERIA ALPHA=0.05 CILEVEL=95. |
| Resources | Processor Time | 00:00:00.23 |
|  | Elapsed Time | 00:00:00.17 |

**null : null**


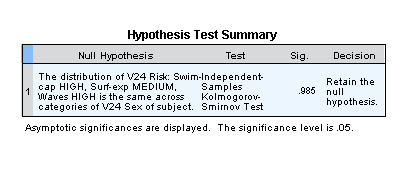


*Nonparametric Tests: Independent Samples.

NPTESTS

/INDEPENDENT TEST (V25.A3.B3.C1) GROUP (V25.sex) KOLMOGOROV_SMIRNOV

/MISSING SCOPE=ANALYSIS USERMISSING=EXCLUDE

/CRITERIA ALPHA=0.05 CILEVEL=95.

**Nonparametric Tests**

| **Notes** | | |
| --- | --- | --- |
| Output Created | | 13-SEP-2019 08:59:46 |
| Comments | |  |
| Input | Data | C:\Users\damianm\2018\Specialist ratings.sav |
|  | Active Dataset | DataSet1 |
|  | Filter | <none> |
|  | Weight | <none> |
|  | Split File | <none> |
|  | N of Rows in Working Data File | 36 |
| Syntax | | NPTESTS  /INDEPENDENT TEST (V25.A3.B3.C1) GROUP (V25.sex) KOLMOGOROV_SMIRNOV  /MISSING SCOPE=ANALYSIS USERMISSING=EXCLUDE  /CRITERIA ALPHA=0.05 CILEVEL=95. |
| Resources | Processor Time | 00:00:00.19 |
|  | Elapsed Time | 00:00:00.17 |

**null : null**


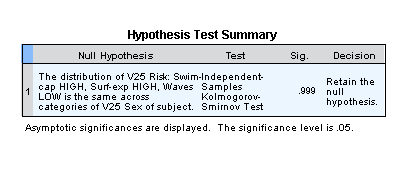


*Nonparametric Tests: Independent Samples.

NPTESTS

/INDEPENDENT TEST (V26.A3.B3.C2) GROUP (V26.sex) KOLMOGOROV_SMIRNOV

/MISSING SCOPE=ANALYSIS USERMISSING=EXCLUDE

/CRITERIA ALPHA=0.05 CILEVEL=95.

**Nonparametric Tests**

| **Notes** | | |
| --- | --- | --- |
| Output Created | | 13-SEP-2019 09:00:07 |
| Comments | |  |
| Input | Data | C:\Users\damianm\2018\Specialist ratings.sav |
|  | Active Dataset | DataSet1 |
|  | Filter | <none> |
|  | Weight | <none> |
|  | Split File | <none> |
|  | N of Rows in Working Data File | 36 |
| Syntax | | NPTESTS  /INDEPENDENT TEST (V26.A3.B3.C2) GROUP (V26.sex) KOLMOGOROV_SMIRNOV  /MISSING SCOPE=ANALYSIS USERMISSING=EXCLUDE  /CRITERIA ALPHA=0.05 CILEVEL=95. |
| Resources | Processor Time | 00:00:00.22 |
|  | Elapsed Time | 00:00:00.17 |

**null : null**


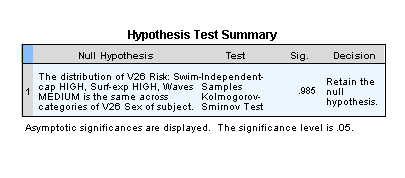


*Nonparametric Tests: Independent Samples.

NPTESTS

/INDEPENDENT TEST (V27.A3.B3.C3) GROUP (V27.sex) KOLMOGOROV_SMIRNOV

/MISSING SCOPE=ANALYSIS USERMISSING=EXCLUDE

/CRITERIA ALPHA=0.05 CILEVEL=95.

**Nonparametric Tests**

| **Notes** | | |
| --- | --- | --- |
| Output Created | | 13-SEP-2019 09:00:26 |
| Comments | |  |
| Input | Data | C:\Users\damianm\2018\Specialist ratings.sav |
|  | Active Dataset | DataSet1 |
|  | Filter | <none> |
|  | Weight | <none> |
|  | Split File | <none> |
|  | N of Rows in Working Data File | 36 |
| Syntax | | NPTESTS  /INDEPENDENT TEST (V27.A3.B3.C3) GROUP (V27.sex) KOLMOGOROV_SMIRNOV  /MISSING SCOPE=ANALYSIS USERMISSING=EXCLUDE  /CRITERIA ALPHA=0.05 CILEVEL=95. |
| Resources | Processor Time | 00:00:00.16 |
|  | Elapsed Time | 00:00:00.15 |

**null : null**


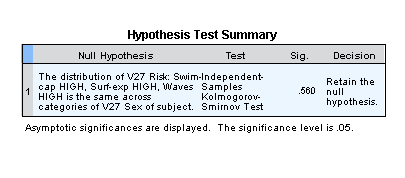


*Nonparametric Tests: Independent Samples.

NPTESTS

/INDEPENDENT TEST (REP.V1.A1.B1.C1) GROUP (REP.V1.sex) KOLMOGOROV_SMIRNOV

/MISSING SCOPE=ANALYSIS USERMISSING=EXCLUDE

/CRITERIA ALPHA=0.05 CILEVEL=95.

**Nonparametric Tests**

| **Notes** | | |
| --- | --- | --- |
| Output Created | | 13-SEP-2019 09:01:24 |
| Comments | |  |
| Input | Data | C:\Users\damianm\2018\Specialist ratings.sav |
|  | Active Dataset | DataSet1 |
|  | Filter | <none> |
|  | Weight | <none> |
|  | Split File | <none> |
|  | N of Rows in Working Data File | 36 |
| Syntax | | NPTESTS  /INDEPENDENT TEST (REP.V1.A1.B1.C1) GROUP (REP.V1.sex) KOLMOGOROV_SMIRNOV  /MISSING SCOPE=ANALYSIS USERMISSING=EXCLUDE  /CRITERIA ALPHA=0.05 CILEVEL=95. |
| Resources | Processor Time | 00:00:00.22 |
|  | Elapsed Time | 00:00:00.17 |

**null : null**


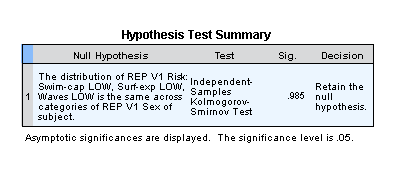


*Nonparametric Tests: Independent Samples.

NPTESTS

/INDEPENDENT TEST (REP.V2.A1.B1.C2) GROUP (REP.V2.sex) KOLMOGOROV_SMIRNOV

/MISSING SCOPE=ANALYSIS USERMISSING=EXCLUDE

/CRITERIA ALPHA=0.05 CILEVEL=95.

**Nonparametric Tests**

| **Notes** | | |
| --- | --- | --- |
| Output Created | | 13-SEP-2019 09:01:46 |
| Comments | |  |
| Input | Data | C:\Users\damianm\2018\Specialist ratings.sav |
|  | Active Dataset | DataSet1 |
|  | Filter | <none> |
|  | Weight | <none> |
|  | Split File | <none> |
|  | N of Rows in Working Data File | 36 |
| Syntax | | NPTESTS  /INDEPENDENT TEST (REP.V2.A1.B1.C2) GROUP (REP.V2.sex) KOLMOGOROV_SMIRNOV  /MISSING SCOPE=ANALYSIS USERMISSING=EXCLUDE  /CRITERIA ALPHA=0.05 CILEVEL=95. |
| Resources | Processor Time | 00:00:00.23 |
|  | Elapsed Time | 00:00:00.19 |

**null : null**


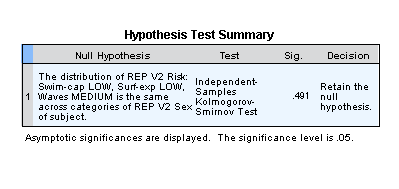


*Nonparametric Tests: Independent Samples.

NPTESTS

/INDEPENDENT TEST (REP.V3.A1.B1.C3) GROUP (REP.V3.sex) KOLMOGOROV_SMIRNOV

/MISSING SCOPE=ANALYSIS USERMISSING=EXCLUDE

/CRITERIA ALPHA=0.05 CILEVEL=95.

**Nonparametric Tests**

| **Notes** | | |
| --- | --- | --- |
| Output Created | | 13-SEP-2019 09:02:07 |
| Comments | |  |
| Input | Data | C:\Users\damianm\2018\Specialist ratings.sav |
|  | Active Dataset | DataSet1 |
|  | Filter | <none> |
|  | Weight | <none> |
|  | Split File | <none> |
|  | N of Rows in Working Data File | 36 |
| Syntax | | NPTESTS  /INDEPENDENT TEST (REP.V3.A1.B1.C3) GROUP (REP.V3.sex) KOLMOGOROV_SMIRNOV  /MISSING SCOPE=ANALYSIS USERMISSING=EXCLUDE  /CRITERIA ALPHA=0.05 CILEVEL=95. |
| Resources | Processor Time | 00:00:00.16 |
|  | Elapsed Time | 00:00:00.13 |

**null : null**


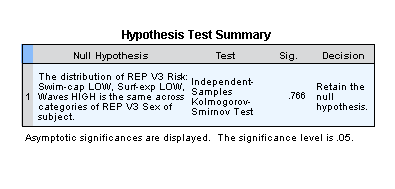


*Nonparametric Tests: Independent Samples.

NPTESTS

/INDEPENDENT TEST (REP.V4.A1.B2.C1) GROUP (REP.V4.sex) KOLMOGOROV_SMIRNOV

/MISSING SCOPE=ANALYSIS USERMISSING=EXCLUDE

/CRITERIA ALPHA=0.05 CILEVEL=95.

**Nonparametric Tests**

| **Notes** | | |
| --- | --- | --- |
| Output Created | | 13-SEP-2019 09:02:25 |
| Comments | |  |
| Input | Data | C:\Users\damianm\2018\Specialist ratings.sav |
|  | Active Dataset | DataSet1 |
|  | Filter | <none> |
|  | Weight | <none> |
|  | Split File | <none> |
|  | N of Rows in Working Data File | 36 |
| Syntax | | NPTESTS  /INDEPENDENT TEST (REP.V4.A1.B2.C1) GROUP (REP.V4.sex) KOLMOGOROV_SMIRNOV  /MISSING SCOPE=ANALYSIS USERMISSING=EXCLUDE  /CRITERIA ALPHA=0.05 CILEVEL=95. |
| Resources | Processor Time | 00:00:00.14 |
|  | Elapsed Time | 00:00:00.15 |

**null : null**


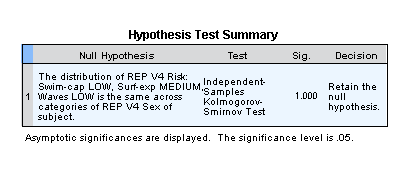


*Nonparametric Tests: Independent Samples.

NPTESTS

/INDEPENDENT TEST (REP.V5.A1.B2.C2) GROUP (REP.V5.sex) KOLMOGOROV_SMIRNOV

/MISSING SCOPE=ANALYSIS USERMISSING=EXCLUDE

/CRITERIA ALPHA=0.05 CILEVEL=95.

**Nonparametric Tests**

| **Notes** | | |
| --- | --- | --- |
| Output Created | | 13-SEP-2019 09:02:43 |
| Comments | |  |
| Input | Data | C:\Users\damianm\2018\Specialist ratings.sav |
|  | Active Dataset | DataSet1 |
|  | Filter | <none> |
|  | Weight | <none> |
|  | Split File | <none> |
|  | N of Rows in Working Data File | 36 |
| Syntax | | NPTESTS  /INDEPENDENT TEST (REP.V5.A1.B2.C2) GROUP (REP.V5.sex) KOLMOGOROV_SMIRNOV  /MISSING SCOPE=ANALYSIS USERMISSING=EXCLUDE  /CRITERIA ALPHA=0.05 CILEVEL=95. |
| Resources | Processor Time | 00:00:00.09 |
|  | Elapsed Time | 00:00:00.19 |

**null : null**


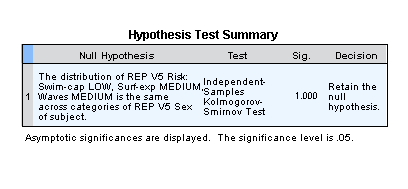


*Nonparametric Tests: Independent Samples.

NPTESTS

/INDEPENDENT TEST (REP.V6.A1.B2.C3) GROUP (REP.V6.sex) KOLMOGOROV_SMIRNOV

/MISSING SCOPE=ANALYSIS USERMISSING=EXCLUDE

/CRITERIA ALPHA=0.05 CILEVEL=95.

**Nonparametric Tests**

| **Notes** | | |
| --- | --- | --- |
| Output Created | | 13-SEP-2019 09:03:02 |
| Comments | |  |
| Input | Data | C:\Users\damianm\2018\Specialist ratings.sav |
|  | Active Dataset | DataSet1 |
|  | Filter | <none> |
|  | Weight | <none> |
|  | Split File | <none> |
|  | N of Rows in Working Data File | 36 |
| Syntax | | NPTESTS  /INDEPENDENT TEST (REP.V6.A1.B2.C3) GROUP (REP.V6.sex) KOLMOGOROV_SMIRNOV  /MISSING SCOPE=ANALYSIS USERMISSING=EXCLUDE  /CRITERIA ALPHA=0.05 CILEVEL=95. |
| Resources | Processor Time | 00:00:00.19 |
|  | Elapsed Time | 00:00:00.27 |

**null : null**


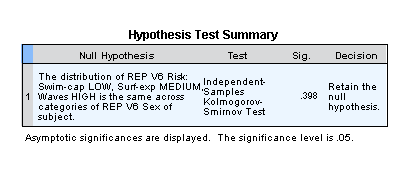


*Nonparametric Tests: Independent Samples.

NPTESTS

/INDEPENDENT TEST (REP.V7.A1.B3.C1) GROUP (REP.V7.sex) KOLMOGOROV_SMIRNOV

/MISSING SCOPE=ANALYSIS USERMISSING=EXCLUDE

/CRITERIA ALPHA=0.05 CILEVEL=95.

**Nonparametric Tests**

| **Notes** | | |
| --- | --- | --- |
| Output Created | | 13-SEP-2019 09:03:23 |
| Comments | |  |
| Input | Data | C:\Users\damianm\2018\Specialist ratings.sav |
|  | Active Dataset | DataSet1 |
|  | Filter | <none> |
|  | Weight | <none> |
|  | Split File | <none> |
|  | N of Rows in Working Data File | 36 |
| Syntax | | NPTESTS  /INDEPENDENT TEST (REP.V7.A1.B3.C1) GROUP (REP.V7.sex) KOLMOGOROV_SMIRNOV  /MISSING SCOPE=ANALYSIS USERMISSING=EXCLUDE  /CRITERIA ALPHA=0.05 CILEVEL=95. |
| Resources | Processor Time | 00:00:00.14 |
|  | Elapsed Time | 00:00:00.14 |

**null : null**


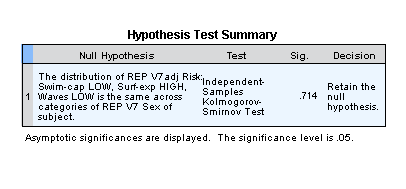


*Nonparametric Tests: Independent Samples.

NPTESTS

/INDEPENDENT TEST (REP.V8.A1.B3.C2) GROUP (REP.V8.sex) KOLMOGOROV_SMIRNOV

/MISSING SCOPE=ANALYSIS USERMISSING=EXCLUDE

/CRITERIA ALPHA=0.05 CILEVEL=95.

**Nonparametric Tests**

| **Notes** | | |
| --- | --- | --- |
| Output Created | | 13-SEP-2019 09:03:58 |
| Comments | |  |
| Input | Data | C:\Users\damianm\2018\Specialist ratings.sav |
|  | Active Dataset | DataSet1 |
|  | Filter | <none> |
|  | Weight | <none> |
|  | Split File | <none> |
|  | N of Rows in Working Data File | 36 |
| Syntax | | NPTESTS  /INDEPENDENT TEST (REP.V8.A1.B3.C2) GROUP (REP.V8.sex) KOLMOGOROV_SMIRNOV  /MISSING SCOPE=ANALYSIS USERMISSING=EXCLUDE  /CRITERIA ALPHA=0.05 CILEVEL=95. |
| Resources | Processor Time | 00:00:00.14 |
|  | Elapsed Time | 00:00:00.13 |

**null : null**


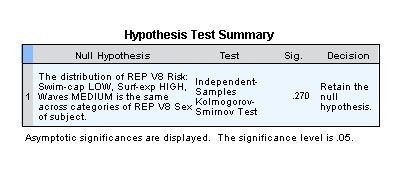


*Nonparametric Tests: Independent Samples.

NPTESTS

/INDEPENDENT TEST (REP.V9.A1.B3.C3) GROUP (REP.V9.sex) KOLMOGOROV_SMIRNOV

/MISSING SCOPE=ANALYSIS USERMISSING=EXCLUDE

/CRITERIA ALPHA=0.05 CILEVEL=95.

**Nonparametric Tests**

| **Notes** | | |
| --- | --- | --- |
| Output Created | | 13-SEP-2019 09:04:30 |
| Comments | |  |
| Input | Data | C:\Users\damianm\2018\Specialist ratings.sav |
|  | Active Dataset | DataSet1 |
|  | Filter | <none> |
|  | Weight | <none> |
|  | Split File | <none> |
|  | N of Rows in Working Data File | 36 |
| Syntax | | NPTESTS  /INDEPENDENT TEST (REP.V9.A1.B3.C3) GROUP (REP.V9.sex) KOLMOGOROV_SMIRNOV  /MISSING SCOPE=ANALYSIS USERMISSING=EXCLUDE  /CRITERIA ALPHA=0.05 CILEVEL=95. |
| Resources | Processor Time | 00:00:00.11 |
|  | Elapsed Time | 00:00:00.14 |

**null : null**


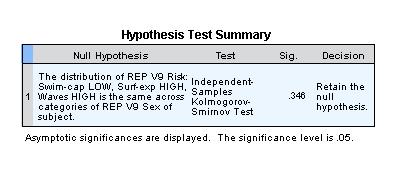


*Nonparametric Tests: Independent Samples.

NPTESTS

/INDEPENDENT TEST (REP.V10.A2.B1.C1) GROUP (REP.V10.sex) KOLMOGOROV_SMIRNOV

/MISSING SCOPE=ANALYSIS USERMISSING=EXCLUDE

/CRITERIA ALPHA=0.05 CILEVEL=95.

**Nonparametric Tests**

| **Notes** | | |
| --- | --- | --- |
| Output Created | | 13-SEP-2019 09:05:09 |
| Comments | |  |
| Input | Data | C:\Users\damianm\2018\Specialist ratings.sav |
|  | Active Dataset | DataSet1 |
|  | Filter | <none> |
|  | Weight | <none> |
|  | Split File | <none> |
|  | N of Rows in Working Data File | 36 |
| Syntax | | NPTESTS  /INDEPENDENT TEST (REP.V10.A2.B1.C1) GROUP (REP.V10.sex) KOLMOGOROV_SMIRNOV  /MISSING SCOPE=ANALYSIS USERMISSING=EXCLUDE  /CRITERIA ALPHA=0.05 CILEVEL=95. |
| Resources | Processor Time | 00:00:00.16 |
|  | Elapsed Time | 00:00:00.13 |

**null : null**


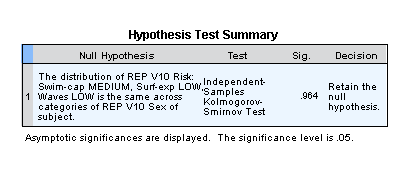


*Nonparametric Tests: Independent Samples.

NPTESTS

/INDEPENDENT TEST (REP.V11.A2.B1.C2) GROUP (REP.V11.sex) KOLMOGOROV_SMIRNOV

/MISSING SCOPE=ANALYSIS USERMISSING=EXCLUDE

/CRITERIA ALPHA=0.05 CILEVEL=95.

**Nonparametric Tests**

| **Notes** | | |
| --- | --- | --- |
| Output Created | | 13-SEP-2019 09:05:54 |
| Comments | |  |
| Input | Data | C:\Users\damianm\2018\Specialist ratings.sav |
|  | Active Dataset | DataSet1 |
|  | Filter | <none> |
|  | Weight | <none> |
|  | Split File | <none> |
|  | N of Rows in Working Data File | 36 |
| Syntax | | NPTESTS  /INDEPENDENT TEST (REP.V11.A2.B1.C2) GROUP (REP.V11.sex) KOLMOGOROV_SMIRNOV  /MISSING SCOPE=ANALYSIS USERMISSING=EXCLUDE  /CRITERIA ALPHA=0.05 CILEVEL=95. |
| Resources | Processor Time | 00:00:00.16 |
|  | Elapsed Time | 00:00:00.13 |

**null : null**


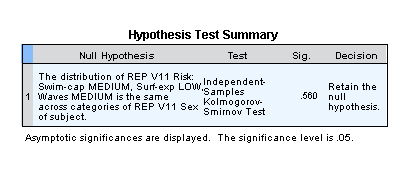


*Nonparametric Tests: Independent Samples.

NPTESTS

/INDEPENDENT TEST (REP.V12.A2.B1.C3) GROUP (REP.V12.sex) KOLMOGOROV_SMIRNOV

/MISSING SCOPE=ANALYSIS USERMISSING=EXCLUDE

/CRITERIA ALPHA=0.05 CILEVEL=95.

**Nonparametric Tests**

| **Notes** | | |
| --- | --- | --- |
| Output Created | | 13-SEP-2019 09:06:12 |
| Comments | |  |
| Input | Data | C:\Users\damianm\2018\Specialist ratings.sav |
|  | Active Dataset | DataSet1 |
|  | Filter | <none> |
|  | Weight | <none> |
|  | Split File | <none> |
|  | N of Rows in Working Data File | 36 |
| Syntax | | NPTESTS  /INDEPENDENT TEST (REP.V12.A2.B1.C3) GROUP (REP.V12.sex) KOLMOGOROV_SMIRNOV  /MISSING SCOPE=ANALYSIS USERMISSING=EXCLUDE  /CRITERIA ALPHA=0.05 CILEVEL=95. |
| Resources | Processor Time | 00:00:00.11 |
|  | Elapsed Time | 00:00:00.14 |

**null : null**


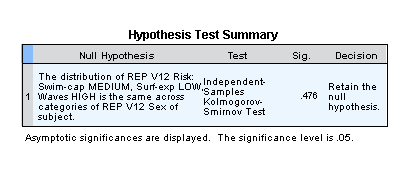


*Nonparametric Tests: Independent Samples.

NPTESTS

/INDEPENDENT TEST (REP.V13.A2.B2.C1) GROUP (REP.V13.sex) KOLMOGOROV_SMIRNOV

/MISSING SCOPE=ANALYSIS USERMISSING=EXCLUDE

/CRITERIA ALPHA=0.05 CILEVEL=95.

**Nonparametric Tests**

| **Notes** | | |
| --- | --- | --- |
| Output Created | | 13-SEP-2019 09:06:33 |
| Comments | |  |
| Input | Data | C:\Users\damianm\2018\Specialist ratings.sav |
|  | Active Dataset | DataSet1 |
|  | Filter | <none> |
|  | Weight | <none> |
|  | Split File | <none> |
|  | N of Rows in Working Data File | 36 |
| Syntax | | NPTESTS  /INDEPENDENT TEST (REP.V13.A2.B2.C1) GROUP (REP.V13.sex) KOLMOGOROV_SMIRNOV  /MISSING SCOPE=ANALYSIS USERMISSING=EXCLUDE  /CRITERIA ALPHA=0.05 CILEVEL=95. |
| Resources | Processor Time | 00:00:00.17 |
|  | Elapsed Time | 00:00:00.14 |

**null : null**


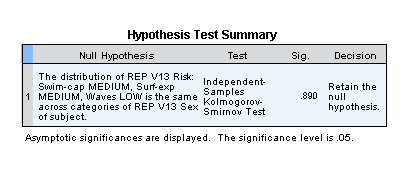


*Nonparametric Tests: Independent Samples.

NPTESTS

/INDEPENDENT TEST (REP.V14.A2.B2.C2) GROUP (REP.V14.sex) KOLMOGOROV_SMIRNOV

/MISSING SCOPE=ANALYSIS USERMISSING=EXCLUDE

/CRITERIA ALPHA=0.05 CILEVEL=95.

**Nonparametric Tests**

| **Notes** | | |
| --- | --- | --- |
| Output Created | | 13-SEP-2019 09:06:52 |
| Comments | |  |
| Input | Data | C:\Users\damianm\2018\Specialist ratings.sav |
|  | Active Dataset | DataSet1 |
|  | Filter | <none> |
|  | Weight | <none> |
|  | Split File | <none> |
|  | N of Rows in Working Data File | 36 |
| Syntax | | NPTESTS  /INDEPENDENT TEST (REP.V14.A2.B2.C2) GROUP (REP.V14.sex) KOLMOGOROV_SMIRNOV  /MISSING SCOPE=ANALYSIS USERMISSING=EXCLUDE  /CRITERIA ALPHA=0.05 CILEVEL=95. |
| Resources | Processor Time | 00:00:00.16 |
|  | Elapsed Time | 00:00:00.13 |

**null : null**


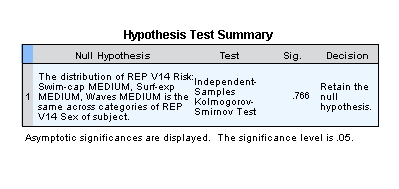


*Nonparametric Tests: Independent Samples.

NPTESTS

/INDEPENDENT TEST (REP.V15.A2.B2.C3) GROUP (REP.V15.sex) KOLMOGOROV_SMIRNOV

/MISSING SCOPE=ANALYSIS USERMISSING=EXCLUDE

/CRITERIA ALPHA=0.05 CILEVEL=95.

**Nonparametric Tests**

| **Notes** | | |
| --- | --- | --- |
| Output Created | | 13-SEP-2019 09:07:09 |
| Comments | |  |
| Input | Data | C:\Users\damianm\2018\Specialist ratings.sav |
|  | Active Dataset | DataSet1 |
|  | Filter | <none> |
|  | Weight | <none> |
|  | Split File | <none> |
|  | N of Rows in Working Data File | 36 |
| Syntax | | NPTESTS  /INDEPENDENT TEST (REP.V15.A2.B2.C3) GROUP (REP.V15.sex) KOLMOGOROV_SMIRNOV  /MISSING SCOPE=ANALYSIS USERMISSING=EXCLUDE  /CRITERIA ALPHA=0.05 CILEVEL=95. |
| Resources | Processor Time | 00:00:00.13 |
|  | Elapsed Time | 00:00:00.16 |

**null : null**


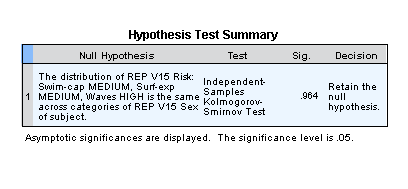


*Nonparametric Tests: Independent Samples.

NPTESTS

/INDEPENDENT TEST (REP.V16.A2.B3.C1) GROUP (REP.V16.sex) KOLMOGOROV_SMIRNOV

/MISSING SCOPE=ANALYSIS USERMISSING=EXCLUDE

/CRITERIA ALPHA=0.05 CILEVEL=95.

**Nonparametric Tests**

| **Notes** | | |
| --- | --- | --- |
| Output Created | | 13-SEP-2019 09:07:28 |
| Comments | |  |
| Input | Data | C:\Users\damianm\2018\Specialist ratings.sav |
|  | Active Dataset | DataSet1 |
|  | Filter | <none> |
|  | Weight | <none> |
|  | Split File | <none> |
|  | N of Rows in Working Data File | 36 |
| Syntax | | NPTESTS  /INDEPENDENT TEST (REP.V16.A2.B3.C1) GROUP (REP.V16.sex) KOLMOGOROV_SMIRNOV  /MISSING SCOPE=ANALYSIS USERMISSING=EXCLUDE  /CRITERIA ALPHA=0.05 CILEVEL=95. |
| Resources | Processor Time | 00:00:00.19 |
|  | Elapsed Time | 00:00:00.15 |

**null : null**


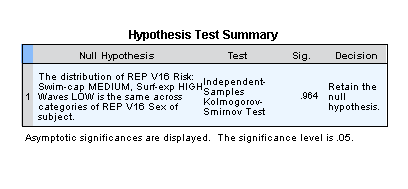


*Nonparametric Tests: Independent Samples.

NPTESTS

/INDEPENDENT TEST (REP.V17.A2.B3.C2) GROUP (REP.V17.sex) KOLMOGOROV_SMIRNOV

/MISSING SCOPE=ANALYSIS USERMISSING=EXCLUDE

/CRITERIA ALPHA=0.05 CILEVEL=95.

**Nonparametric Tests**

| **Notes** | | |
| --- | --- | --- |
| Output Created | | 13-SEP-2019 09:07:45 |
| Comments | |  |
| Input | Data | C:\Users\damianm\2018\Specialist ratings.sav |
|  | Active Dataset | DataSet1 |
|  | Filter | <none> |
|  | Weight | <none> |
|  | Split File | <none> |
|  | N of Rows in Working Data File | 36 |
| Syntax | | NPTESTS  /INDEPENDENT TEST (REP.V17.A2.B3.C2) GROUP (REP.V17.sex) KOLMOGOROV_SMIRNOV  /MISSING SCOPE=ANALYSIS USERMISSING=EXCLUDE  /CRITERIA ALPHA=0.05 CILEVEL=95. |
| Resources | Processor Time | 00:00:00.08 |
|  | Elapsed Time | 00:00:00.16 |

**null : null**


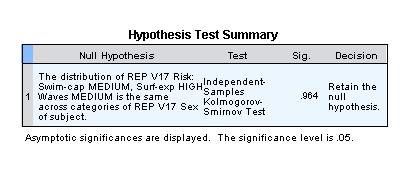


*Nonparametric Tests: Independent Samples.

NPTESTS

/INDEPENDENT TEST (REP.V18.A2.B3.C3) GROUP (REP.V18.sex) KOLMOGOROV_SMIRNOV

/MISSING SCOPE=ANALYSIS USERMISSING=EXCLUDE

/CRITERIA ALPHA=0.05 CILEVEL=95.

**Nonparametric Tests**

| **Notes** | | |
| --- | --- | --- |
| Output Created | | 13-SEP-2019 09:08:05 |
| Comments | |  |
| Input | Data | C:\Users\damianm\2018\Specialist ratings.sav |
|  | Active Dataset | DataSet1 |
|  | Filter | <none> |
|  | Weight | <none> |
|  | Split File | <none> |
|  | N of Rows in Working Data File | 36 |
| Syntax | | NPTESTS  /INDEPENDENT TEST (REP.V18.A2.B3.C3) GROUP (REP.V18.sex) KOLMOGOROV_SMIRNOV  /MISSING SCOPE=ANALYSIS USERMISSING=EXCLUDE  /CRITERIA ALPHA=0.05 CILEVEL=95. |
| Resources | Processor Time | 00:00:00.09 |
|  | Elapsed Time | 00:00:00.15 |

**null : null**


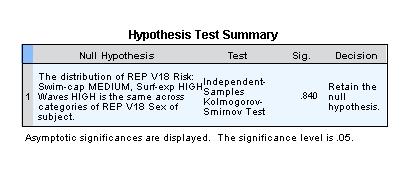


*Nonparametric Tests: Independent Samples.

NPTESTS

/INDEPENDENT TEST (REP.V19.A3.B1.C1) GROUP (REP.V19.sex) KOLMOGOROV_SMIRNOV

/MISSING SCOPE=ANALYSIS USERMISSING=EXCLUDE

/CRITERIA ALPHA=0.05 CILEVEL=95.

**Nonparametric Tests**

| **Notes** | | |
| --- | --- | --- |
| Output Created | | 13-SEP-2019 09:08:22 |
| Comments | |  |
| Input | Data | C:\Users\damianm\2018\Specialist ratings.sav |
|  | Active Dataset | DataSet1 |
|  | Filter | <none> |
|  | Weight | <none> |
|  | Split File | <none> |
|  | N of Rows in Working Data File | 36 |
| Syntax | | NPTESTS  /INDEPENDENT TEST (REP.V19.A3.B1.C1) GROUP (REP.V19.sex) KOLMOGOROV_SMIRNOV  /MISSING SCOPE=ANALYSIS USERMISSING=EXCLUDE  /CRITERIA ALPHA=0.05 CILEVEL=95. |
| Resources | Processor Time | 00:00:00.13 |
|  | Elapsed Time | 00:00:00.14 |

**null : null**


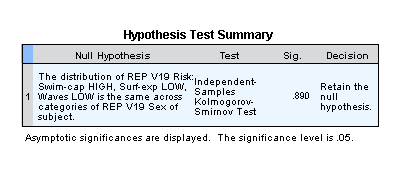


*Nonparametric Tests: Independent Samples.

NPTESTS

/INDEPENDENT TEST (REP.V20.A3.B1.C2) GROUP (REP.V20.sex) KOLMOGOROV_SMIRNOV

/MISSING SCOPE=ANALYSIS USERMISSING=EXCLUDE

/CRITERIA ALPHA=0.05 CILEVEL=95.

**Nonparametric Tests**

| **Notes** | | |
| --- | --- | --- |
| Output Created | | 13-SEP-2019 09:08:42 |
| Comments | |  |
| Input | Data | C:\Users\damianm\2018\Specialist ratings.sav |
|  | Active Dataset | DataSet1 |
|  | Filter | <none> |
|  | Weight | <none> |
|  | Split File | <none> |
|  | N of Rows in Working Data File | 36 |
| Syntax | | NPTESTS  /INDEPENDENT TEST (REP.V20.A3.B1.C2) GROUP (REP.V20.sex) KOLMOGOROV_SMIRNOV  /MISSING SCOPE=ANALYSIS USERMISSING=EXCLUDE  /CRITERIA ALPHA=0.05 CILEVEL=95. |
| Resources | Processor Time | 00:00:00.11 |
|  | Elapsed Time | 00:00:00.13 |

**null : null**


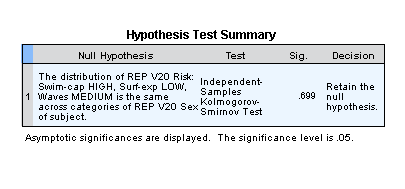


*Nonparametric Tests: Independent Samples.

NPTESTS

/INDEPENDENT TEST (REP.V21.A3.B1.C3) GROUP (REP.V21.sex) KOLMOGOROV_SMIRNOV

/MISSING SCOPE=ANALYSIS USERMISSING=EXCLUDE

/CRITERIA ALPHA=0.05 CILEVEL=95.

**Nonparametric Tests**

| **Notes** | | |
| --- | --- | --- |
| Output Created | | 13-SEP-2019 09:09:07 |
| Comments | |  |
| Input | Data | C:\Users\damianm\2018\Specialist ratings.sav |
|  | Active Dataset | DataSet1 |
|  | Filter | <none> |
|  | Weight | <none> |
|  | Split File | <none> |
|  | N of Rows in Working Data File | 36 |
| Syntax | | NPTESTS  /INDEPENDENT TEST (REP.V21.A3.B1.C3) GROUP (REP.V21.sex) KOLMOGOROV_SMIRNOV  /MISSING SCOPE=ANALYSIS USERMISSING=EXCLUDE  /CRITERIA ALPHA=0.05 CILEVEL=95. |
| Resources | Processor Time | 00:00:00.09 |
|  | Elapsed Time | 00:00:00.13 |

**null : null**


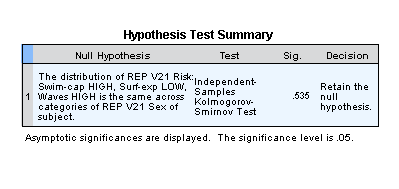


*Nonparametric Tests: Independent Samples.

NPTESTS

/INDEPENDENT TEST (REP.V22.A3.B2.C1) GROUP (REP.V22.sex) KOLMOGOROV_SMIRNOV

/MISSING SCOPE=ANALYSIS USERMISSING=EXCLUDE

/CRITERIA ALPHA=0.05 CILEVEL=95.

**Nonparametric Tests**

| **Notes** | | |
| --- | --- | --- |
| Output Created | | 13-SEP-2019 09:09:27 |
| Comments | |  |
| Input | Data | C:\Users\damianm\2018\Specialist ratings.sav |
|  | Active Dataset | DataSet1 |
|  | Filter | <none> |
|  | Weight | <none> |
|  | Split File | <none> |
|  | N of Rows in Working Data File | 36 |
| Syntax | | NPTESTS  /INDEPENDENT TEST (REP.V22.A3.B2.C1) GROUP (REP.V22.sex) KOLMOGOROV_SMIRNOV  /MISSING SCOPE=ANALYSIS USERMISSING=EXCLUDE  /CRITERIA ALPHA=0.05 CILEVEL=95. |
| Resources | Processor Time | 00:00:00.16 |
|  | Elapsed Time | 00:00:00.16 |

**null : null**


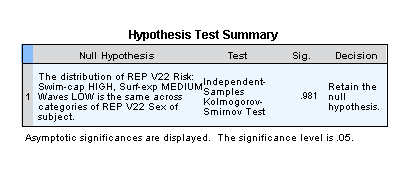


*Nonparametric Tests: Independent Samples.

NPTESTS

/INDEPENDENT TEST (REP.V23.A3.B2.C2) GROUP (REP.V23.sex) KOLMOGOROV_SMIRNOV

/MISSING SCOPE=ANALYSIS USERMISSING=EXCLUDE

/CRITERIA ALPHA=0.05 CILEVEL=95.

**Nonparametric Tests**

| **Notes** | | |
| --- | --- | --- |
| Output Created | | 13-SEP-2019 09:09:49 |
| Comments | |  |
| Input | Data | C:\Users\damianm\2018\Specialist ratings.sav |
|  | Active Dataset | DataSet1 |
|  | Filter | <none> |
|  | Weight | <none> |
|  | Split File | <none> |
|  | N of Rows in Working Data File | 36 |
| Syntax | | NPTESTS  /INDEPENDENT TEST (REP.V23.A3.B2.C2) GROUP (REP.V23.sex) KOLMOGOROV_SMIRNOV  /MISSING SCOPE=ANALYSIS USERMISSING=EXCLUDE  /CRITERIA ALPHA=0.05 CILEVEL=95. |
| Resources | Processor Time | 00:00:00.30 |
|  | Elapsed Time | 00:00:00.20 |

**null : null**


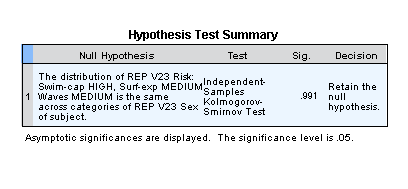


*Nonparametric Tests: Independent Samples.

NPTESTS

/INDEPENDENT TEST (REP.V24.A3.B2.C3) GROUP (REP.V24.sex) KOLMOGOROV_SMIRNOV

/MISSING SCOPE=ANALYSIS USERMISSING=EXCLUDE

/CRITERIA ALPHA=0.05 CILEVEL=95.

**Nonparametric Tests**

| **Notes** | | |
| --- | --- | --- |
| Output Created | | 13-SEP-2019 09:10:09 |
| Comments | |  |
| Input | Data | C:\Users\damianm\2018\Specialist ratings.sav |
|  | Active Dataset | DataSet1 |
|  | Filter | <none> |
|  | Weight | <none> |
|  | Split File | <none> |
|  | N of Rows in Working Data File | 36 |
| Syntax | | NPTESTS  /INDEPENDENT TEST (REP.V24.A3.B2.C3) GROUP (REP.V24.sex) KOLMOGOROV_SMIRNOV  /MISSING SCOPE=ANALYSIS USERMISSING=EXCLUDE  /CRITERIA ALPHA=0.05 CILEVEL=95. |
| Resources | Processor Time | 00:00:00.17 |
|  | Elapsed Time | 00:00:00.16 |

**null : null**


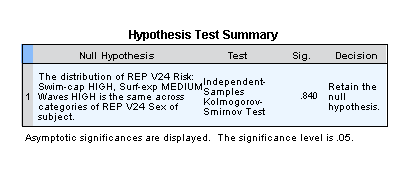


*Nonparametric Tests: Independent Samples.

NPTESTS

/INDEPENDENT TEST (REP.V25.A3.B3.C1) GROUP (REP.V25.sex) KOLMOGOROV_SMIRNOV

/MISSING SCOPE=ANALYSIS USERMISSING=EXCLUDE

/CRITERIA ALPHA=0.05 CILEVEL=95.

**Nonparametric Tests**

| **Notes** | | |
| --- | --- | --- |
| Output Created | | 13-SEP-2019 09:10:29 |
| Comments | |  |
| Input | Data | C:\Users\damianm\2018\Specialist ratings.sav |
|  | Active Dataset | DataSet1 |
|  | Filter | <none> |
|  | Weight | <none> |
|  | Split File | <none> |
|  | N of Rows in Working Data File | 36 |
| Syntax | | NPTESTS  /INDEPENDENT TEST (REP.V25.A3.B3.C1) GROUP (REP.V25.sex) KOLMOGOROV_SMIRNOV  /MISSING SCOPE=ANALYSIS USERMISSING=EXCLUDE  /CRITERIA ALPHA=0.05 CILEVEL=95. |
| Resources | Processor Time | 00:00:00.13 |
|  | Elapsed Time | 00:00:00.13 |

**null : null**


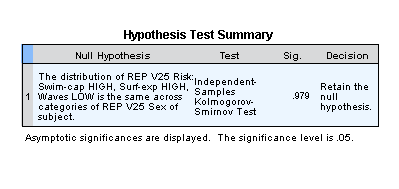


*Nonparametric Tests: Independent Samples.

NPTESTS

/INDEPENDENT TEST (REP.V26.A3.B3.C2) GROUP (REP.V26.sex) KOLMOGOROV_SMIRNOV

/MISSING SCOPE=ANALYSIS USERMISSING=EXCLUDE

/CRITERIA ALPHA=0.05 CILEVEL=95.

**Nonparametric Tests**

| **Notes** | | |
| --- | --- | --- |
| Output Created | | 13-SEP-2019 09:10:48 |
| Comments | |  |
| Input | Data | C:\Users\damianm\2018\Specialist ratings.sav |
|  | Active Dataset | DataSet1 |
|  | Filter | <none> |
|  | Weight | <none> |
|  | Split File | <none> |
|  | N of Rows in Working Data File | 36 |
| Syntax | | NPTESTS  /INDEPENDENT TEST (REP.V26.A3.B3.C2) GROUP (REP.V26.sex) KOLMOGOROV_SMIRNOV  /MISSING SCOPE=ANALYSIS USERMISSING=EXCLUDE  /CRITERIA ALPHA=0.05 CILEVEL=95. |
| Resources | Processor Time | 00:00:00.06 |
|  | Elapsed Time | 00:00:00.14 |

**null : null**


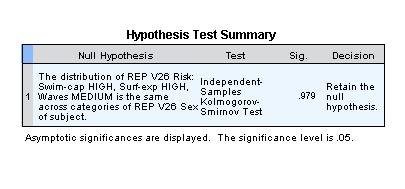


| **Notes** | | |
| --- | --- | --- |
| Output Created | | 13-SEP-2019 09:11:07 |
| Comments | |  |
| Input | Data | C:\Users\damianm\2018\Specialist ratings.sav |
|  | Active Dataset | DataSet1 |
|  | Filter | <none> |
|  | Weight | <none> |
|  | Split File | <none> |
|  | N of Rows in Working Data File | 36 |
| Syntax | | NPTESTS  /INDEPENDENT TEST (REP.V27.A3.B3.C3) GROUP (REP.V27.sex) KOLMOGOROV_SMIRNOV  /MISSING SCOPE=ANALYSIS USERMISSING=EXCLUDE  /CRITERIA ALPHA=0.05 CILEVEL=95. |
| Resources | Processor Time | 00:00:00.14 |
|  | Elapsed Time | 00:00:00.13 |

| **Notes** | | |
| --- | --- | --- |
| Output Created | | 13-SEP-2019 09:15:40 |
| Comments | |  |
| Input | Data | C:\Users\damianm\2018\Specialist ratings.sav |
|  | Active Dataset | DataSet1 |
|  | Filter | <none> |
|  | Weight | <none> |
|  | Split File | <none> |
|  | N of Rows in Working Data File | 36 |
| Syntax | | NPTESTS  /INDEPENDENT TEST (V10.A2.B1.C1) GROUP (V10.sex) KOLMOGOROV_SMIRNOV  /MISSING SCOPE=ANALYSIS USERMISSING=EXCLUDE  /CRITERIA ALPHA=0.05 CILEVEL=95. |
| Resources | Processor Time | 00:00:00.13 |
|  | Elapsed Time | 00:00:00.13 |

| **Notes** | | |
| --- | --- | --- |
| Output Created | | 13-SEP-2019 09:19:22 |
| Comments | |  |
| Input | Data | C:\Users\damianm\2018\Specialist ratings.sav |
|  | Active Dataset | DataSet1 |
|  | Filter | <none> |
|  | Weight | <none> |
|  | Split File | <none> |
|  | N of Rows in Working Data File | 36 |
| Syntax | | NPTESTS  /RELATED TEST(V1.A1.B1.C1 REP.V1.A1.B1.C1) WILCOXON  /MISSING SCOPE=ANALYSIS USERMISSING=EXCLUDE  /CRITERIA ALPHA=0.002 CILEVEL=95. |
| Resources | Processor Time | 00:00:00.14 |
|  | Elapsed Time | 00:00:00.13 |

*Nonparametric Tests: Related Samples.

NPTESTS

/RELATED TEST(V1.A1.B1.C1 REP.V1.A1.B1.C1) WILCOXON

/MISSING SCOPE=ANALYSIS USERMISSING=EXCLUDE

/CRITERIA ALPHA=0.05 CILEVEL=95.

**Nonparametric Tests**

| **Notes** | | |
| --- | --- | --- |
| Output Created | | 13-SEP-2019 09:21:33 |
| Comments | |  |
| Input | Data | C:\Users\damianm\2018\Specialist ratings.sav |
|  | Active Dataset | DataSet1 |
|  | Filter | <none> |
|  | Weight | <none> |
|  | Split File | <none> |
|  | N of Rows in Working Data File | 36 |
| Syntax | | NPTESTS  /RELATED TEST(V1.A1.B1.C1 REP.V1.A1.B1.C1) WILCOXON  /MISSING SCOPE=ANALYSIS USERMISSING=EXCLUDE  /CRITERIA ALPHA=0.05 CILEVEL=95. |
| Resources | Processor Time | 00:00:00.13 |
|  | Elapsed Time | 00:00:00.12 |

**null : null**


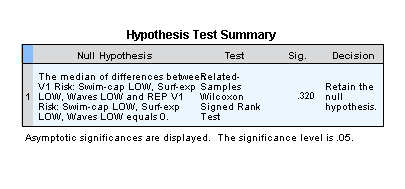


*Nonparametric Tests: Related Samples.

NPTESTS

/RELATED TEST(V2.A1.B1.C2 REP.V2.A1.B1.C2) WILCOXON

/MISSING SCOPE=ANALYSIS USERMISSING=EXCLUDE

/CRITERIA ALPHA=0.05 CILEVEL=95.

**Nonparametric Tests**

| **Notes** | | |
| --- | --- | --- |
| Output Created | | 13-SEP-2019 09:22:26 |
| Comments | |  |
| Input | Data | C:\Users\damianm\2018\Specialist ratings.sav |
|  | Active Dataset | DataSet1 |
|  | Filter | <none> |
|  | Weight | <none> |
|  | Split File | <none> |
|  | N of Rows in Working Data File | 36 |
| Syntax | | NPTESTS  /RELATED TEST(V2.A1.B1.C2 REP.V2.A1.B1.C2) WILCOXON  /MISSING SCOPE=ANALYSIS USERMISSING=EXCLUDE  /CRITERIA ALPHA=0.05 CILEVEL=95. |
| Resources | Processor Time | 00:00:00.13 |
|  | Elapsed Time | 00:00:00.13 |

**null : null**


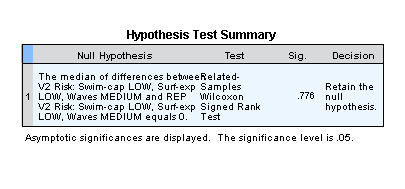


*Nonparametric Tests: Related Samples.

NPTESTS

/RELATED TEST(V3.A1.B1.C3 REP.V3.A1.B1.C3) WILCOXON

/MISSING SCOPE=ANALYSIS USERMISSING=EXCLUDE

/CRITERIA ALPHA=0.05 CILEVEL=95.

**Nonparametric Tests**

| **Notes** | | |
| --- | --- | --- |
| Output Created | | 13-SEP-2019 09:22:57 |
| Comments | |  |
| Input | Data | C:\Users\damianm\2018\Specialist ratings.sav |
|  | Active Dataset | DataSet1 |
|  | Filter | <none> |
|  | Weight | <none> |
|  | Split File | <none> |
|  | N of Rows in Working Data File | 36 |
| Syntax | | NPTESTS  /RELATED TEST(V3.A1.B1.C3 REP.V3.A1.B1.C3) WILCOXON  /MISSING SCOPE=ANALYSIS USERMISSING=EXCLUDE  /CRITERIA ALPHA=0.05 CILEVEL=95. |
| Resources | Processor Time | 00:00:00.08 |
|  | Elapsed Time | 00:00:00.12 |

**null : null**


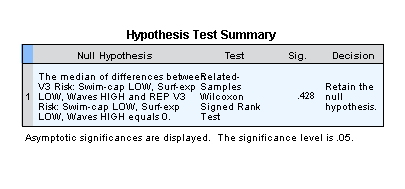


*Nonparametric Tests: Related Samples.

NPTESTS

/RELATED TEST(V4.A1.B2.C1 REP.V4.A1.B2.C1) WILCOXON

/MISSING SCOPE=ANALYSIS USERMISSING=EXCLUDE

/CRITERIA ALPHA=0.05 CILEVEL=95.

**Nonparametric Tests**

| **Notes** | | |
| --- | --- | --- |
| Output Created | | 13-SEP-2019 09:23:34 |
| Comments | |  |
| Input | Data | C:\Users\damianm\2018\Specialist ratings.sav |
|  | Active Dataset | DataSet1 |
|  | Filter | <none> |
|  | Weight | <none> |
|  | Split File | <none> |
|  | N of Rows in Working Data File | 36 |
| Syntax | | NPTESTS  /RELATED TEST(V4.A1.B2.C1 REP.V4.A1.B2.C1) WILCOXON  /MISSING SCOPE=ANALYSIS USERMISSING=EXCLUDE  /CRITERIA ALPHA=0.05 CILEVEL=95. |
| Resources | Processor Time | 00:00:00.11 |
|  | Elapsed Time | 00:00:00.14 |

**null : null**


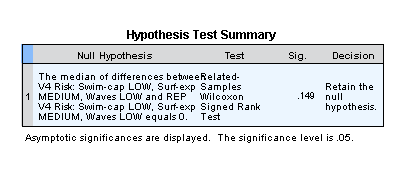


*Nonparametric Tests: Related Samples.

NPTESTS

/RELATED TEST(V5.A1.B2.C2 REP.V5.A1.B2.C2) WILCOXON

/MISSING SCOPE=ANALYSIS USERMISSING=EXCLUDE

/CRITERIA ALPHA=0.05 CILEVEL=95.

**Nonparametric Tests**

| **Notes** | | |
| --- | --- | --- |
| Output Created | | 13-SEP-2019 09:23:56 |
| Comments | |  |
| Input | Data | C:\Users\damianm\2018\Specialist ratings.sav |
|  | Active Dataset | DataSet1 |
|  | Filter | <none> |
|  | Weight | <none> |
|  | Split File | <none> |
|  | N of Rows in Working Data File | 36 |
| Syntax | | NPTESTS  /RELATED TEST(V5.A1.B2.C2 REP.V5.A1.B2.C2) WILCOXON  /MISSING SCOPE=ANALYSIS USERMISSING=EXCLUDE  /CRITERIA ALPHA=0.05 CILEVEL=95. |
| Resources | Processor Time | 00:00:00.14 |
|  | Elapsed Time | 00:00:00.15 |

**null : null**


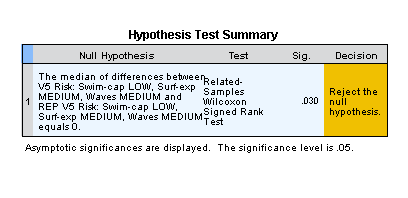


*Nonparametric Tests: Related Samples.

NPTESTS

/RELATED TEST(V6.A1.B2.C3 REP.V6.A1.B2.C3) WILCOXON

/MISSING SCOPE=ANALYSIS USERMISSING=EXCLUDE

/CRITERIA ALPHA=0.05 CILEVEL=95.

**Nonparametric Tests**

| **Notes** | | |
| --- | --- | --- |
| Output Created | | 13-SEP-2019 09:24:15 |
| Comments | |  |
| Input | Data | C:\Users\damianm\2018\Specialist ratings.sav |
|  | Active Dataset | DataSet1 |
|  | Filter | <none> |
|  | Weight | <none> |
|  | Split File | <none> |
|  | N of Rows in Working Data File | 36 |
| Syntax | | NPTESTS  /RELATED TEST(V6.A1.B2.C3 REP.V6.A1.B2.C3) WILCOXON  /MISSING SCOPE=ANALYSIS USERMISSING=EXCLUDE  /CRITERIA ALPHA=0.05 CILEVEL=95. |
| Resources | Processor Time | 00:00:00.08 |
|  | Elapsed Time | 00:00:00.14 |

**null : null**


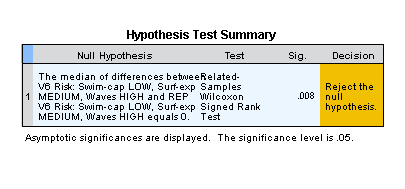


*Nonparametric Tests: Related Samples.

NPTESTS

/RELATED TEST(V7.A1.B3.C1 REP.V7.A1.B3.C1) WILCOXON

/MISSING SCOPE=ANALYSIS USERMISSING=EXCLUDE

/CRITERIA ALPHA=0.05 CILEVEL=95.

**Nonparametric Tests**

| **Notes** | | |
| --- | --- | --- |
| Output Created | | 13-SEP-2019 09:24:45 |
| Comments | |  |
| Input | Data | C:\Users\damianm\2018\Specialist ratings.sav |
|  | Active Dataset | DataSet1 |
|  | Filter | <none> |
|  | Weight | <none> |
|  | Split File | <none> |
|  | N of Rows in Working Data File | 36 |
| Syntax | | NPTESTS  /RELATED TEST(V7.A1.B3.C1 REP.V7.A1.B3.C1) WILCOXON  /MISSING SCOPE=ANALYSIS USERMISSING=EXCLUDE  /CRITERIA ALPHA=0.05 CILEVEL=95. |
| Resources | Processor Time | 00:00:00.13 |
|  | Elapsed Time | 00:00:00.13 |

**null : null**


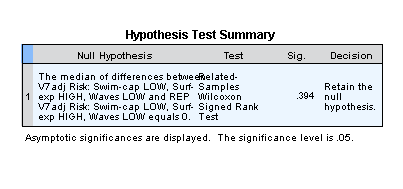


*Nonparametric Tests: Related Samples.

NPTESTS

/RELATED TEST(V8.A1.B3.C2 REP.V8.A1.B3.C2) WILCOXON

/MISSING SCOPE=ANALYSIS USERMISSING=EXCLUDE

/CRITERIA ALPHA=0.05 CILEVEL=95.

**Nonparametric Tests**

| **Notes** | | |
| --- | --- | --- |
| Output Created | | 13-SEP-2019 09:25:15 |
| Comments | |  |
| Input | Data | C:\Users\damianm\2018\Specialist ratings.sav |
|  | Active Dataset | DataSet1 |
|  | Filter | <none> |
|  | Weight | <none> |
|  | Split File | <none> |
|  | N of Rows in Working Data File | 36 |
| Syntax | | NPTESTS  /RELATED TEST(V8.A1.B3.C2 REP.V8.A1.B3.C2) WILCOXON  /MISSING SCOPE=ANALYSIS USERMISSING=EXCLUDE  /CRITERIA ALPHA=0.05 CILEVEL=95. |
| Resources | Processor Time | 00:00:00.13 |
|  | Elapsed Time | 00:00:00.13 |

**null : null**


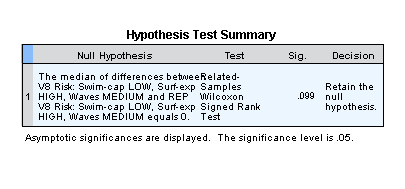


*Nonparametric Tests: Related Samples.

NPTESTS

/RELATED TEST(V9.A1.B3.C3 REP.V9.A1.B3.C3) WILCOXON

/MISSING SCOPE=ANALYSIS USERMISSING=EXCLUDE

/CRITERIA ALPHA=0.05 CILEVEL=95.

**Nonparametric Tests**

| **Notes** | | |
| --- | --- | --- |
| Output Created | | 13-SEP-2019 09:25:51 |
| Comments | |  |
| Input | Data | C:\Users\damianm\2018\Specialist ratings.sav |
|  | Active Dataset | DataSet1 |
|  | Filter | <none> |
|  | Weight | <none> |
|  | Split File | <none> |
|  | N of Rows in Working Data File | 36 |
| Syntax | | NPTESTS  /RELATED TEST(V9.A1.B3.C3 REP.V9.A1.B3.C3) WILCOXON  /MISSING SCOPE=ANALYSIS USERMISSING=EXCLUDE  /CRITERIA ALPHA=0.05 CILEVEL=95. |
| Resources | Processor Time | 00:00:00.13 |
|  | Elapsed Time | 00:00:00.14 |

**null : null**


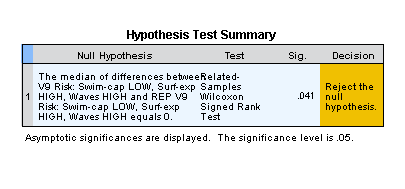


*Nonparametric Tests: Related Samples.

NPTESTS

/RELATED TEST(V10.A2.B1.C1 REP.V10.A2.B1.C1) WILCOXON

/MISSING SCOPE=ANALYSIS USERMISSING=EXCLUDE

/CRITERIA ALPHA=0.05 CILEVEL=95.

**Nonparametric Tests**

| **Notes** | | |
| --- | --- | --- |
| Output Created | | 13-SEP-2019 09:26:14 |
| Comments | |  |
| Input | Data | C:\Users\damianm\2018\Specialist ratings.sav |
|  | Active Dataset | DataSet1 |
|  | Filter | <none> |
|  | Weight | <none> |
|  | Split File | <none> |
|  | N of Rows in Working Data File | 36 |
| Syntax | | NPTESTS  /RELATED TEST(V10.A2.B1.C1 REP.V10.A2.B1.C1) WILCOXON  /MISSING SCOPE=ANALYSIS USERMISSING=EXCLUDE  /CRITERIA ALPHA=0.05 CILEVEL=95. |
| Resources | Processor Time | 00:00:00.14 |
|  | Elapsed Time | 00:00:00.12 |

**null : null**


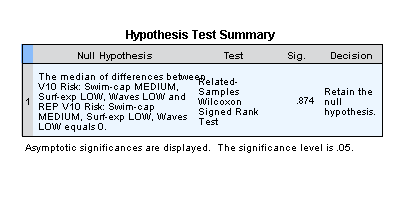


*Nonparametric Tests: Related Samples.

NPTESTS

/RELATED TEST(V11.A2.B1.C2 REP.V11.A2.B1.C2) WILCOXON

/MISSING SCOPE=ANALYSIS USERMISSING=EXCLUDE

/CRITERIA ALPHA=0.05 CILEVEL=95.

**Nonparametric Tests**

| **Notes** | | |
| --- | --- | --- |
| Output Created | | 13-SEP-2019 09:26:31 |
| Comments | |  |
| Input | Data | C:\Users\damianm\2018\Specialist ratings.sav |
|  | Active Dataset | DataSet1 |
|  | Filter | <none> |
|  | Weight | <none> |
|  | Split File | <none> |
|  | N of Rows in Working Data File | 36 |
| Syntax | | NPTESTS  /RELATED TEST(V11.A2.B1.C2 REP.V11.A2.B1.C2) WILCOXON  /MISSING SCOPE=ANALYSIS USERMISSING=EXCLUDE  /CRITERIA ALPHA=0.05 CILEVEL=95. |
| Resources | Processor Time | 00:00:00.11 |
|  | Elapsed Time | 00:00:00.15 |

**null : null**


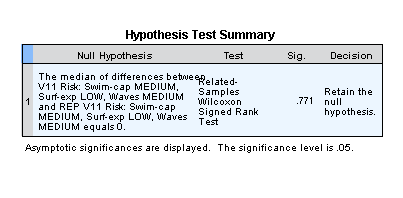


*Nonparametric Tests: Related Samples.

NPTESTS

/RELATED TEST(V12.A2.B1.C3 REP.V12.A2.B1.C3) WILCOXON

/MISSING SCOPE=ANALYSIS USERMISSING=EXCLUDE

/CRITERIA ALPHA=0.05 CILEVEL=95.

**Nonparametric Tests**

| **Notes** | | |
| --- | --- | --- |
| Output Created | | 13-SEP-2019 09:26:55 |
| Comments | |  |
| Input | Data | C:\Users\damianm\2018\Specialist ratings.sav |
|  | Active Dataset | DataSet1 |
|  | Filter | <none> |
|  | Weight | <none> |
|  | Split File | <none> |
|  | N of Rows in Working Data File | 36 |
| Syntax | | NPTESTS  /RELATED TEST(V12.A2.B1.C3 REP.V12.A2.B1.C3) WILCOXON  /MISSING SCOPE=ANALYSIS USERMISSING=EXCLUDE  /CRITERIA ALPHA=0.05 CILEVEL=95. |
| Resources | Processor Time | 00:00:00.13 |
|  | Elapsed Time | 00:00:00.14 |

**null : null**


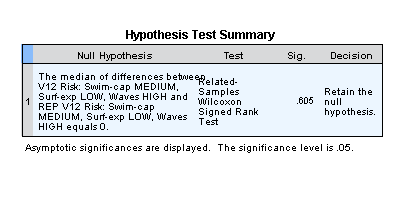


*Nonparametric Tests: Related Samples.

NPTESTS

/RELATED TEST(V13.A2.B2.C1 REP.V13.A2.B2.C1) WILCOXON

/MISSING SCOPE=ANALYSIS USERMISSING=EXCLUDE

/CRITERIA ALPHA=0.05 CILEVEL=95.

**Nonparametric Tests**

| **Notes** | | |
| --- | --- | --- |
| Output Created | | 13-SEP-2019 09:27:12 |
| Comments | |  |
| Input | Data | C:\Users\damianm\2018\Specialist ratings.sav |
|  | Active Dataset | DataSet1 |
|  | Filter | <none> |
|  | Weight | <none> |
|  | Split File | <none> |
|  | N of Rows in Working Data File | 36 |
| Syntax | | NPTESTS  /RELATED TEST(V13.A2.B2.C1 REP.V13.A2.B2.C1) WILCOXON  /MISSING SCOPE=ANALYSIS USERMISSING=EXCLUDE  /CRITERIA ALPHA=0.05 CILEVEL=95. |
| Resources | Processor Time | 00:00:00.14 |
|  | Elapsed Time | 00:00:00.14 |

**null : null**


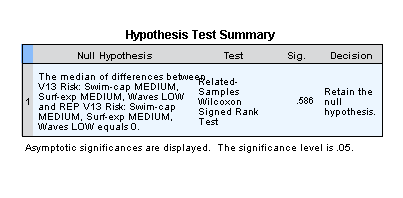


*Nonparametric Tests: Related Samples.

NPTESTS

/RELATED TEST(V14.A2.B2.C2 REP.V14.A2.B2.C2) WILCOXON

/MISSING SCOPE=ANALYSIS USERMISSING=EXCLUDE

/CRITERIA ALPHA=0.05 CILEVEL=95.

**Nonparametric Tests**

| **Notes** | | |
| --- | --- | --- |
| Output Created | | 13-SEP-2019 09:27:36 |
| Comments | |  |
| Input | Data | C:\Users\damianm\2018\Specialist ratings.sav |
|  | Active Dataset | DataSet1 |
|  | Filter | <none> |
|  | Weight | <none> |
|  | Split File | <none> |
|  | N of Rows in Working Data File | 36 |
| Syntax | | NPTESTS  /RELATED TEST(V14.A2.B2.C2 REP.V14.A2.B2.C2) WILCOXON  /MISSING SCOPE=ANALYSIS USERMISSING=EXCLUDE  /CRITERIA ALPHA=0.05 CILEVEL=95. |
| Resources | Processor Time | 00:00:00.13 |
|  | Elapsed Time | 00:00:00.13 |

**null : null**


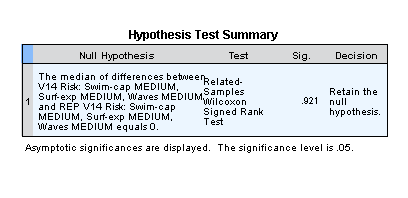


*Nonparametric Tests: Related Samples.

NPTESTS

/RELATED TEST(V15.A2.B2.C3 REP.V15.A2.B2.C3) WILCOXON

/MISSING SCOPE=ANALYSIS USERMISSING=EXCLUDE

/CRITERIA ALPHA=0.05 CILEVEL=95.

**Nonparametric Tests**

| **Notes** | | |
| --- | --- | --- |
| Output Created | | 13-SEP-2019 09:28:01 |
| Comments | |  |
| Input | Data | C:\Users\damianm\2018\Specialist ratings.sav |
|  | Active Dataset | DataSet1 |
|  | Filter | <none> |
|  | Weight | <none> |
|  | Split File | <none> |
|  | N of Rows in Working Data File | 36 |
| Syntax | | NPTESTS  /RELATED TEST(V15.A2.B2.C3 REP.V15.A2.B2.C3) WILCOXON  /MISSING SCOPE=ANALYSIS USERMISSING=EXCLUDE  /CRITERIA ALPHA=0.05 CILEVEL=95. |
| Resources | Processor Time | 00:00:00.14 |
|  | Elapsed Time | 00:00:00.17 |

**null : null**


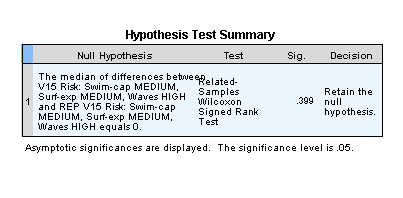


*Nonparametric Tests: Related Samples.

NPTESTS

/RELATED TEST(V16.A2.B3.C1 REP.V16.A2.B3.C1) WILCOXON

/MISSING SCOPE=ANALYSIS USERMISSING=EXCLUDE

/CRITERIA ALPHA=0.05 CILEVEL=95.

**Nonparametric Tests**

| **Notes** | | |
| --- | --- | --- |
| Output Created | | 13-SEP-2019 09:28:18 |
| Comments | |  |
| Input | Data | C:\Users\damianm\2018\Specialist ratings.sav |
|  | Active Dataset | DataSet1 |
|  | Filter | <none> |
|  | Weight | <none> |
|  | Split File | <none> |
|  | N of Rows in Working Data File | 36 |
| Syntax | | NPTESTS  /RELATED TEST(V16.A2.B3.C1 REP.V16.A2.B3.C1) WILCOXON  /MISSING SCOPE=ANALYSIS USERMISSING=EXCLUDE  /CRITERIA ALPHA=0.05 CILEVEL=95. |
| Resources | Processor Time | 00:00:00.06 |
|  | Elapsed Time | 00:00:00.13 |

**null : null**


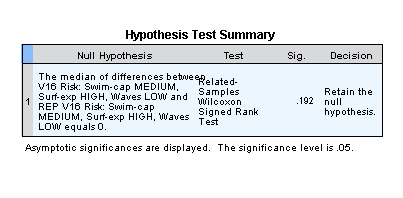


*Nonparametric Tests: Related Samples.

NPTESTS

/RELATED TEST(V17.A2.B3.C2 REP.V17.A2.B3.C2) WILCOXON

/MISSING SCOPE=ANALYSIS USERMISSING=EXCLUDE

/CRITERIA ALPHA=0.05 CILEVEL=95.

**Nonparametric Tests**

| **Notes** | | |
| --- | --- | --- |
| Output Created | | 13-SEP-2019 09:28:38 |
| Comments | |  |
| Input | Data | C:\Users\damianm\2018\Specialist ratings.sav |
|  | Active Dataset | DataSet1 |
|  | Filter | <none> |
|  | Weight | <none> |
|  | Split File | <none> |
|  | N of Rows in Working Data File | 36 |
| Syntax | | NPTESTS  /RELATED TEST(V17.A2.B3.C2 REP.V17.A2.B3.C2) WILCOXON  /MISSING SCOPE=ANALYSIS USERMISSING=EXCLUDE  /CRITERIA ALPHA=0.05 CILEVEL=95. |
| Resources | Processor Time | 00:00:00.17 |
|  | Elapsed Time | 00:00:00.15 |

**null : null**


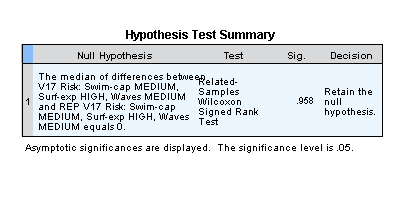


*Nonparametric Tests: Related Samples.

NPTESTS

/RELATED TEST(V18.A2.B3.C3 REP.V18.A2.B3.C3) WILCOXON

/MISSING SCOPE=ANALYSIS USERMISSING=EXCLUDE

/CRITERIA ALPHA=0.05 CILEVEL=95.

**Nonparametric Tests**

| **Notes** | | |
| --- | --- | --- |
| Output Created | | 13-SEP-2019 09:28:57 |
| Comments | |  |
| Input | Data | C:\Users\damianm\2018\Specialist ratings.sav |
|  | Active Dataset | DataSet1 |
|  | Filter | <none> |
|  | Weight | <none> |
|  | Split File | <none> |
|  | N of Rows in Working Data File | 36 |
| Syntax | | NPTESTS  /RELATED TEST(V18.A2.B3.C3 REP.V18.A2.B3.C3) WILCOXON  /MISSING SCOPE=ANALYSIS USERMISSING=EXCLUDE  /CRITERIA ALPHA=0.05 CILEVEL=95. |
| Resources | Processor Time | 00:00:00.14 |
|  | Elapsed Time | 00:00:00.15 |

**null : null**


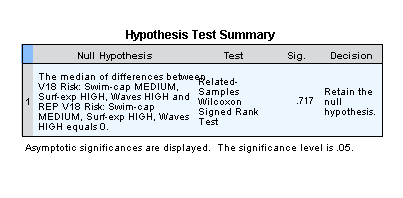


*Nonparametric Tests: Related Samples.

NPTESTS

/RELATED TEST(V19.A3.B1.C1 REP.V19.A3.B1.C1) WILCOXON

/MISSING SCOPE=ANALYSIS USERMISSING=EXCLUDE

/CRITERIA ALPHA=0.05 CILEVEL=95.

**Nonparametric Tests**

| **Notes** | | |
| --- | --- | --- |
| Output Created | | 13-SEP-2019 09:29:17 |
| Comments | |  |
| Input | Data | C:\Users\damianm\2018\Specialist ratings.sav |
|  | Active Dataset | DataSet1 |
|  | Filter | <none> |
|  | Weight | <none> |
|  | Split File | <none> |
|  | N of Rows in Working Data File | 36 |
| Syntax | | NPTESTS  /RELATED TEST(V19.A3.B1.C1 REP.V19.A3.B1.C1) WILCOXON  /MISSING SCOPE=ANALYSIS USERMISSING=EXCLUDE  /CRITERIA ALPHA=0.05 CILEVEL=95. |
| Resources | Processor Time | 00:00:00.09 |
|  | Elapsed Time | 00:00:00.16 |

**null : null**


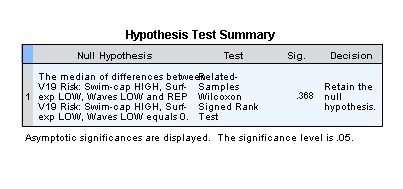


*Nonparametric Tests: Related Samples.

NPTESTS

/RELATED TEST(V20.A3.B1.C2 REP.V20.A3.B1.C2) WILCOXON

/MISSING SCOPE=ANALYSIS USERMISSING=EXCLUDE

/CRITERIA ALPHA=0.05 CILEVEL=95.

**Nonparametric Tests**

| **Notes** | | |
| --- | --- | --- |
| Output Created | | 13-SEP-2019 09:29:35 |
| Comments | |  |
| Input | Data | C:\Users\damianm\2018\Specialist ratings.sav |
|  | Active Dataset | DataSet1 |
|  | Filter | <none> |
|  | Weight | <none> |
|  | Split File | <none> |
|  | N of Rows in Working Data File | 36 |
| Syntax | | NPTESTS  /RELATED TEST(V20.A3.B1.C2 REP.V20.A3.B1.C2) WILCOXON  /MISSING SCOPE=ANALYSIS USERMISSING=EXCLUDE  /CRITERIA ALPHA=0.05 CILEVEL=95. |
| Resources | Processor Time | 00:00:00.09 |
|  | Elapsed Time | 00:00:00.12 |

**null : null**


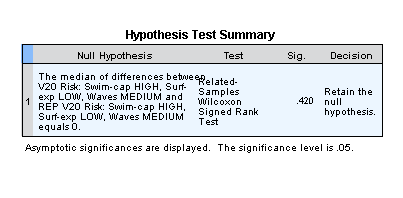


*Nonparametric Tests: Related Samples.

NPTESTS

/RELATED TEST(V21.A3.B1.C3 REP.V21.A3.B1.C3) WILCOXON

/MISSING SCOPE=ANALYSIS USERMISSING=EXCLUDE

/CRITERIA ALPHA=0.05 CILEVEL=95.

**Nonparametric Tests**

| **Notes** | | |
| --- | --- | --- |
| Output Created | | 13-SEP-2019 09:29:50 |
| Comments | |  |
| Input | Data | C:\Users\damianm\2018\Specialist ratings.sav |
|  | Active Dataset | DataSet1 |
|  | Filter | <none> |
|  | Weight | <none> |
|  | Split File | <none> |
|  | N of Rows in Working Data File | 36 |
| Syntax | | NPTESTS  /RELATED TEST(V21.A3.B1.C3 REP.V21.A3.B1.C3) WILCOXON  /MISSING SCOPE=ANALYSIS USERMISSING=EXCLUDE  /CRITERIA ALPHA=0.05 CILEVEL=95. |
| Resources | Processor Time | 00:00:00.13 |
|  | Elapsed Time | 00:00:00.14 |

**null : null**


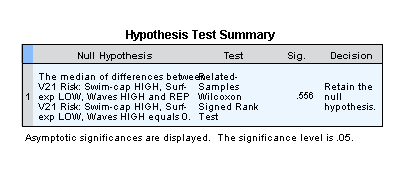


*Nonparametric Tests: Related Samples.

NPTESTS

/RELATED TEST(V22.A3.B2.C1 REP.V22.A3.B2.C1) WILCOXON

/MISSING SCOPE=ANALYSIS USERMISSING=EXCLUDE

/CRITERIA ALPHA=0.05 CILEVEL=95.

**Nonparametric Tests**

| **Notes** | | |
| --- | --- | --- |
| Output Created | | 13-SEP-2019 09:30:10 |
| Comments | |  |
| Input | Data | C:\Users\damianm\2018\Specialist ratings.sav |
|  | Active Dataset | DataSet1 |
|  | Filter | <none> |
|  | Weight | <none> |
|  | Split File | <none> |
|  | N of Rows in Working Data File | 36 |
| Syntax | | NPTESTS  /RELATED TEST(V22.A3.B2.C1 REP.V22.A3.B2.C1) WILCOXON  /MISSING SCOPE=ANALYSIS USERMISSING=EXCLUDE  /CRITERIA ALPHA=0.05 CILEVEL=95. |
| Resources | Processor Time | 00:00:00.13 |
|  | Elapsed Time | 00:00:00.25 |

**null : null**


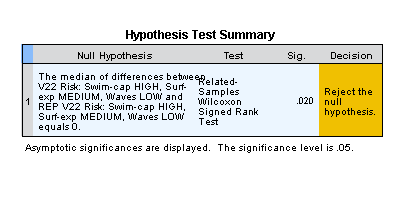


*Nonparametric Tests: Related Samples.

NPTESTS

/RELATED TEST(V23.A3.B2.C2 REP.V23.A3.B2.C2) WILCOXON

/MISSING SCOPE=ANALYSIS USERMISSING=EXCLUDE

/CRITERIA ALPHA=0.05 CILEVEL=95.

**Nonparametric Tests**

| **Notes** | | |
| --- | --- | --- |
| Output Created | | 13-SEP-2019 09:30:29 |
| Comments | |  |
| Input | Data | C:\Users\damianm\2018\Specialist ratings.sav |
|  | Active Dataset | DataSet1 |
|  | Filter | <none> |
|  | Weight | <none> |
|  | Split File | <none> |
|  | N of Rows in Working Data File | 36 |
| Syntax | | NPTESTS  /RELATED TEST(V23.A3.B2.C2 REP.V23.A3.B2.C2) WILCOXON  /MISSING SCOPE=ANALYSIS USERMISSING=EXCLUDE  /CRITERIA ALPHA=0.05 CILEVEL=95. |
| Resources | Processor Time | 00:00:00.13 |
|  | Elapsed Time | 00:00:00.13 |

**null : null**


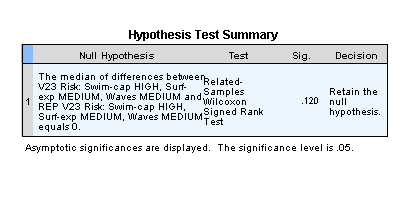


*Nonparametric Tests: Related Samples.

NPTESTS

/RELATED TEST(V24.A3.B2.C3 REP.V24.A3.B2.C3) WILCOXON

/MISSING SCOPE=ANALYSIS USERMISSING=EXCLUDE

/CRITERIA ALPHA=0.05 CILEVEL=95.

**Nonparametric Tests**

| **Notes** | | |
| --- | --- | --- |
| Output Created | | 13-SEP-2019 09:30:46 |
| Comments | |  |
| Input | Data | C:\Users\damianm\2018\Specialist ratings.sav |
|  | Active Dataset | DataSet1 |
|  | Filter | <none> |
|  | Weight | <none> |
|  | Split File | <none> |
|  | N of Rows in Working Data File | 36 |
| Syntax | | NPTESTS  /RELATED TEST(V24.A3.B2.C3 REP.V24.A3.B2.C3) WILCOXON  /MISSING SCOPE=ANALYSIS USERMISSING=EXCLUDE  /CRITERIA ALPHA=0.05 CILEVEL=95. |
| Resources | Processor Time | 00:00:00.14 |
|  | Elapsed Time | 00:00:00.15 |

**null : null**


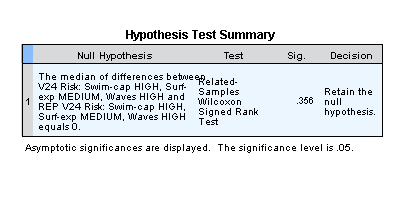


*Nonparametric Tests: Related Samples.

NPTESTS

/RELATED TEST(V25.A3.B3.C1 REP.V25.A3.B3.C1) WILCOXON

/MISSING SCOPE=ANALYSIS USERMISSING=EXCLUDE

/CRITERIA ALPHA=0.05 CILEVEL=95.

**Nonparametric Tests**

| **Notes** | | |
| --- | --- | --- |
| Output Created | | 13-SEP-2019 09:31:03 |
| Comments | |  |
| Input | Data | C:\Users\damianm\2018\Specialist ratings.sav |
|  | Active Dataset | DataSet1 |
|  | Filter | <none> |
|  | Weight | <none> |
|  | Split File | <none> |
|  | N of Rows in Working Data File | 36 |
| Syntax | | NPTESTS  /RELATED TEST(V25.A3.B3.C1 REP.V25.A3.B3.C1) WILCOXON  /MISSING SCOPE=ANALYSIS USERMISSING=EXCLUDE  /CRITERIA ALPHA=0.05 CILEVEL=95. |
| Resources | Processor Time | 00:00:00.11 |
|  | Elapsed Time | 00:00:00.14 |

**null : null**


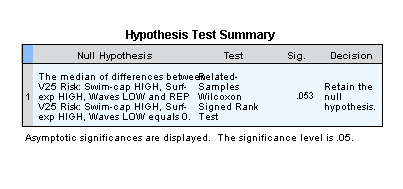


*Nonparametric Tests: Related Samples.

NPTESTS

/RELATED TEST(V26.A3.B3.C2 REP.V26.A3.B3.C2) WILCOXON

/MISSING SCOPE=ANALYSIS USERMISSING=EXCLUDE

/CRITERIA ALPHA=0.05 CILEVEL=95.

**Nonparametric Tests**

| **Notes** | | |
| --- | --- | --- |
| Output Created | | 13-SEP-2019 09:31:19 |
| Comments | |  |
| Input | Data | C:\Users\damianm\2018\Specialist ratings.sav |
|  | Active Dataset | DataSet1 |
|  | Filter | <none> |
|  | Weight | <none> |
|  | Split File | <none> |
|  | N of Rows in Working Data File | 36 |
| Syntax | | NPTESTS  /RELATED TEST(V26.A3.B3.C2 REP.V26.A3.B3.C2) WILCOXON  /MISSING SCOPE=ANALYSIS USERMISSING=EXCLUDE  /CRITERIA ALPHA=0.05 CILEVEL=95. |
| Resources | Processor Time | 00:00:00.14 |
|  | Elapsed Time | 00:00:00.13 |

**null : null**


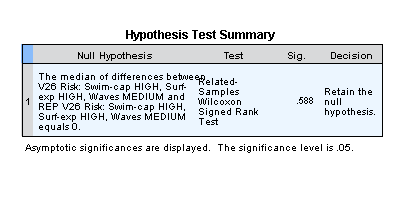


*Nonparametric Tests: Related Samples.

NPTESTS

/RELATED TEST(V27.A3.B3.C3 REP.V27.A3.B3.C3) WILCOXON

/MISSING SCOPE=ANALYSIS USERMISSING=EXCLUDE

/CRITERIA ALPHA=0.05 CILEVEL=95.

**Nonparametric Tests**

| **Notes** | | |
| --- | --- | --- |
| Output Created | | 13-SEP-2019 09:31:42 |
| Comments | |  |
| Input | Data | C:\Users\damianm\2018\Specialist ratings.sav |
|  | Active Dataset | DataSet1 |
|  | Filter | <none> |
|  | Weight | <none> |
|  | Split File | <none> |
|  | N of Rows in Working Data File | 36 |
| Syntax | | NPTESTS  /RELATED TEST(V27.A3.B3.C3 REP.V27.A3.B3.C3) WILCOXON  /MISSING SCOPE=ANALYSIS USERMISSING=EXCLUDE  /CRITERIA ALPHA=0.05 CILEVEL=95. |
| Resources | Processor Time | 00:00:00.09 |
|  | Elapsed Time | 00:00:00.14 |

**null : null**


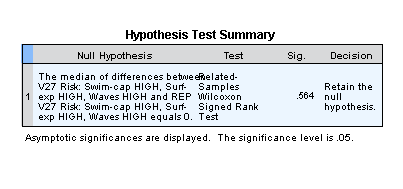

Supplement: S1 Data Output — (DOCX) [file pone.0211166.s005.docx]
